# Influence of CCL2-mediated modulation of ALIX in the budding and replication of viruses from multiple families

**DOI:** 10.1128/mbio.02241-25

**Published:** 2025-09-25

**Authors:** Dina Mofed, Anjali Gowripalan, Jacob Berrigan, Pratyush Kumar Das, Nivedita Pujari, David Ajasin, Swati Haldar, John McCullough, Yongwei Zhang, Ganjam V. Kalpana, Anne Bresnick, Margaret Kielian, Duncan W. Wilson, Jinghang Zhang, Kartik Chandran, Vinayaka R. Prasad

**Affiliations:** 1 Department of Microbiology and Immunology, Albert Einstein College of Medicine 2006, Bronx, New York, USA; 2 Department of Cell Biology, Albert Einstein College of Medicine 2006, Bronx, New York, USA; 3 Department of Developmental and Molecular Biology, Albert Einstein College of Medicine 2006, Bronx, New York, USA; 4 Department of Genetics, Albert Einstein College of Medicine 2006, Bronx, New York, USA; 5 Department of Biochemistry, School of Medicine, University of Utah 7060, Salt Lake City, Utah, USA; 6 Department of Biochemistry, Albert Einstein College of Medicine 2006, Bronx, New York, USA; 7 Dominick P. Purpura Department of Neuroscience, Albert Einstein College of Medicine 2006, Bronx, New York, USA; Massachusetts Institute of Technology, Cambridge, Massachusetts, USA

**Keywords:** human immunodeficiency virus, virus budding, HIV replication, CCL2, ESCRT, ALIX

## Abstract

Signaling by C-C motif ligand 2 (CCL2), a β-chemokine, modulates HIV-1 budding and release by mobilizing ALG-2-interacting protein X (ALIX) from the F-actin cytoskeleton to the cytosol. Immunodepleting CCL2 in the medium sequesters ALIX to F-actin. We developed a novel tool to study HIV budding and release without mutating viral late domains or silencing ESCRT genes, but by blocking CCL2 signaling using CRISPR-Cas9 knockout (KO) of the CCL2 or CCR2 genes. We knocked out CCL2 (CCL2KO) and CCR2 (CCR2KO) singly or together (double knockout) in HeLa cells and confirmed that knockout was associated with the absence of CCL2 or CCR2 expression. In KO cells, ALIX was associated with the F-actin cytoskeleton, while in control cells, it was associated with the cytosolic soluble fraction. In KO cells, HIV-1 production was profoundly reduced (10-fold). Strikingly, for CCL2KO cells, the addition of CCL2 mobilized ALIX to the soluble fraction, and virus production was stimulated to levels higher than those of untreated HeLa cells. We utilized these cells to test the involvement of ALIX in the budding and/or replication of several viruses, including Simian Immunodeficiency Virus (SIV), Equine Infectious Anemia Virus (EIAV), Herpes Simplex Virus type 1 (HSV-1), Dengue virus (DENV), and Hazara virus (HAZV). Budding and release of SIV and EIAV were both inhibited in CCL2KO cells and rescued by CCL2 addition. Replication of HSV-1 and DENV was unaffected in CCL2KO cells, confirming that ALIX is not involved in their replication. Finally, HAZV replication was affected by CCL2 signaling. Our studies indicate that CCL2 signaling and ALIX mobilization are important for several viral families.

**IMPORTANCE:**

C-C motif ligand 2 (CCL2) plays a regulatory role in the budding and release of HIV-1 in macrophages and HeLa cells. CCL2 signaling mobilizes ALG-2-interacting protein X (ALIX) from the F-actin cytoskeleton to the soluble cytosol, where it is accessible for recruitment by the HIV-1 Gag polyprotein in the assembling virions at the plasma membrane. In previous studies, CCL2 immunodepletion, which blocks CCL2 signaling, resulted in ALIX sequestration to the F-actin cytoskeleton and inhibited virus production. Here, we developed a HeLa CCL2 gene knockout cell line and found that abrogation of CCL2 signaling can be restored by CCL2 addition, as evidenced by the restoration of ALIX to the cytosolic fraction and rescue of HIV-1 release. Employing such a system, we tested Simian Immunodeficiency Virus, Equine Infectious Anemia Virus, Herpes Simplex Virus type 1, Dengue, and Hazara virus for their dependence on ALIX for virus replication. The results indicate that CCL2 signaling and ALIX release from F-actin may play a role in the replication of several viruses.

## INTRODUCTION

HIV-1 replication consists of a series of coordinated events beginning with virus attachment and engagement with the CD4 receptor and a coreceptor (CCR5 or CXCR4), followed by fusion and entry, reverse transcription, nuclear transport, uncoating, integration, transcription, translation, virus assembly, and release. In general, successive events appear to be triggered by the formation of the previous intermediate in a highly conducive cellular environment. Very few steps in viral replication are extrinsically regulated with the exception of LTR transcription from an integrated provirus, which can be modulated by mitogens such as phytohemagglutinin or phorbol 12-myristic 13-acetate (1). Until recently, extrinsic agents that modulate late events in HIV-1 replication were unknown. We reported that, in HIV-infected macrophages and HeLa cells, C-C motif ligand 2 (CCL2/MCP-1) mobilizes ALG-2-interacting protein X (ALIX) from the F-actin cytoskeletal networks to the soluble cytosol, where it can be efficiently recruited by assembling virus particles, resulting in a higher efficiency of virus production (2). This responsiveness to the presence of extracellular CCL2 required the presence of the late domain motif LYPX in HIV-1 Gag p6 protein, which recruits ALIX protein to the budding virion particles at the plasma membrane. ALIX, in turn, recruits CHMP4B, a member of the Endosomal Sorting Complexes Required for Transport (ESCRT-III) family of proteins, which catalyzes the final membrane severing step at the bud neck. HIV-1 possesses an additional late motif, PTAP, which also facilitates CHMP4B recruitment but via an ALIX-independent pathway. The ability of the LYPX motif to bind ALIX is maximized in the presence of CCL2 signaling, thus leading to a higher efficiency of budding and improved virus fitness (2).

CCL2 is one of the many pro-inflammatory cytokines and chemokines induced upon HIV-1 infection (3, 4). In HIV-1-infected individuals, plasma CCL2 levels are elevated in viremic people living with HIV but not in virologically suppressed individuals (5). Other groups have tested the effect of blocking CCL2 signaling on HIV-1 replication in macrophages. They showed that neutralizing anti-CCL2 antibodies, which block CCL2 from binding to its receptor, CCR2, inhibited virus replication and that the virus capsid (CA; p24) protein accumulated intracellularly (6). We reported that CCL2 does not affect the replication of HIV-1C, as this subtype of HIV-1 viruses lacks the LYPX motif in Gag-p6 due to the deletion of a LY dipeptide (2). This finding was further confirmed utilizing an HIV-1_IndieC1_-mGreenLantern reporter virus (7). We showed that the presence of a functional LYPX late motif in the virus and expression of ALIX in the producer cell were both necessary for the HIV-1 subtype B virus to display enhanced production in response to CCL2 levels in the medium. Our results suggest that viruses with the ability to utilize ALIX for their replication can exploit a pro-inflammatory environment (via secreted CCL2) to increase their release and replication fitness. Inserting the LY dipeptide sequence to recreate a functional LYPX motif rendered the HIV-1C virus responsive to CCL2 (2). Furthermore, variant subtype C viruses have emerged that have acquired an LYPX-like motif, which restored the ability to bind to ALIX, increasing fitness and the ability to respond to CCL2 (2).

The HIV-1 Gag protein is necessary for all key processes of virus assembly, budding, and release (8). The process underlying HIV-1 budding and release is a complex interplay of many factors, such as viral proteins, cellular lipids, and small molecules. HIV-1 budding is orchestrated by the coordinated action of the members of the ESCRT family of proteins. Cells normally employ the ESCRT machinery to effect a wide range of membrane remodeling and fission events, including cytokinetic abscission, post-mitotic nuclear membrane sealing, and intraluminal vesicle formation at endosomes (9–12). The recruitment of ESCRT proteins by the Gag protein within the assembling virus particles is a crucial initial step in viral budding (12). The ESCRT recruitment begins when the PTAP motif in the p6 domain of Gag binds to the Ubiquitin E2 Variant domain of Tumor Susceptibility Gene 101 (TSG101), a component of ESCRT-I. The ESCRT I complex then recruits the ESCRT III proteins CHMP4B, CHMP3, and CHMP2A. While it has also been reported that ESCRT-II is required for PTAP-mediated HIV-1 budding (13), it is generally viewed as largely dispensable for HIV-1 budding (14, 15). Separately, the LYPX motif, also in p6, recruits ALIX, an accessory ESCRT factor, which can directly recruit CHMP4B in an ESCRT II-independent manner. This provides an alternative pathway for ESCRT III recruitment (16, 17). The ESCRT machinery also orchestrates the budding of other retroviruses, such as the Simian Immunodeficiency Virus (SIV) (18), Equine Infectious Anemia Virus (EIAV) (19), Murine Leukemia Virus (MuLV) (20), Human T-cell Leukemia Virus Type 1, Rous Sarcoma Virus (RSV) (21), and Foamy viruses (22). The recruitment of ESCRT components via Gag late domains is a conserved strategy among retroviruses.

The main goal of this study was to develop novel tools to study the pathways and mechanisms of HIV budding that involve the LYPX late domain of Gag and the ALIX protein. Previously, it has been possible to mutate late domains (LYPX, PTAP, or PPxY) or silence host proteins (ALIX or TSG101) to specifically inhibit each of these pathways and study the impact on virion budding. However, there have not been attempts to genetically ablate CCL2 or CCR2 in the producer cells to abrogate the LYPX-ALIX pathway of budding. Importantly, no reversible cell system, such as a CCL2 gene knockout, has been reported, in which the CCL2-signaling blockade can be rescued just by adding CCL2 to the medium. Therefore, we used CRISPR/Cas9 technology to develop knockout (KO) cell lines that are deficient in the expression of CCL2, CCR2, or both proteins. We have verified the lack of expression and/or production of these proteins and tested these cells for their ability to support the replication and release of viruses via the LYPX-ALIX and/or PTAP-TSG101 pathways. Furthermore, we have used CCL2KO cell lines to investigate whether the CCL2-mediated regulation of virus production is important for other enveloped viruses such as SIV, EIAV, HSV-1, Dengue (DENV), the Nairovirus, and Hazara virus (HAZV). Our results show that (i) CCL2-ALIX signaling axis also exists in other lentiviruses such as SIV and EIAV; (ii) neither HSV-1 nor DENV uses ALIX for their replication; and (iii) HAZV replication uses ALIX for its replication and that the CCL2-ALIX signaling axis has an influence on its replication levels. Thus, it appears that many viruses other than HIV-1 can also improve their replication fitness under pro-inflammatory conditions.

## RESULTS

### Generation of CRISPR-Cas9 gene knockout cell lines

To produce CCL2KO cell lines, HeLa cell clones that were transduced with the CRISPR-Cas9 cassette and gRNAs, and the transduced cells were screened by sequencing the genomic regions surrounding each gene’s CRISPR target site(s). Sequencing analysis showed that small indels or fragment excisions were present at or near all CRISPR target sites, which indicated efficient CRISPR-NHEJ-mediated gene modification (Fig. S1 to S4). We generated four CCL2KO, three CCR2KO, and three CCL2/CCR2 double KO (DKO) cell lines. Of these, two CCL2KO cell lines showed leaky expression and thus were not pursued further. Phenotypically, the KO cell lines showed minor but noticeable morphological changes in comparison with the parental HeLa cells. Specifically, all three types of KO cell lines tended to appear slightly smaller and less elongated. Notably, a subset of DKO cells, in particular, displayed a more spherical morphology. Moreover, the KO cell lines generally grew more slowly and took approximately 1–2 days longer to reach confluence under standard culture conditions compared to parental HeLa cells (data not shown).

### Expression of CCL2 and CCR2 in KO cell lines

**Fig 1:**
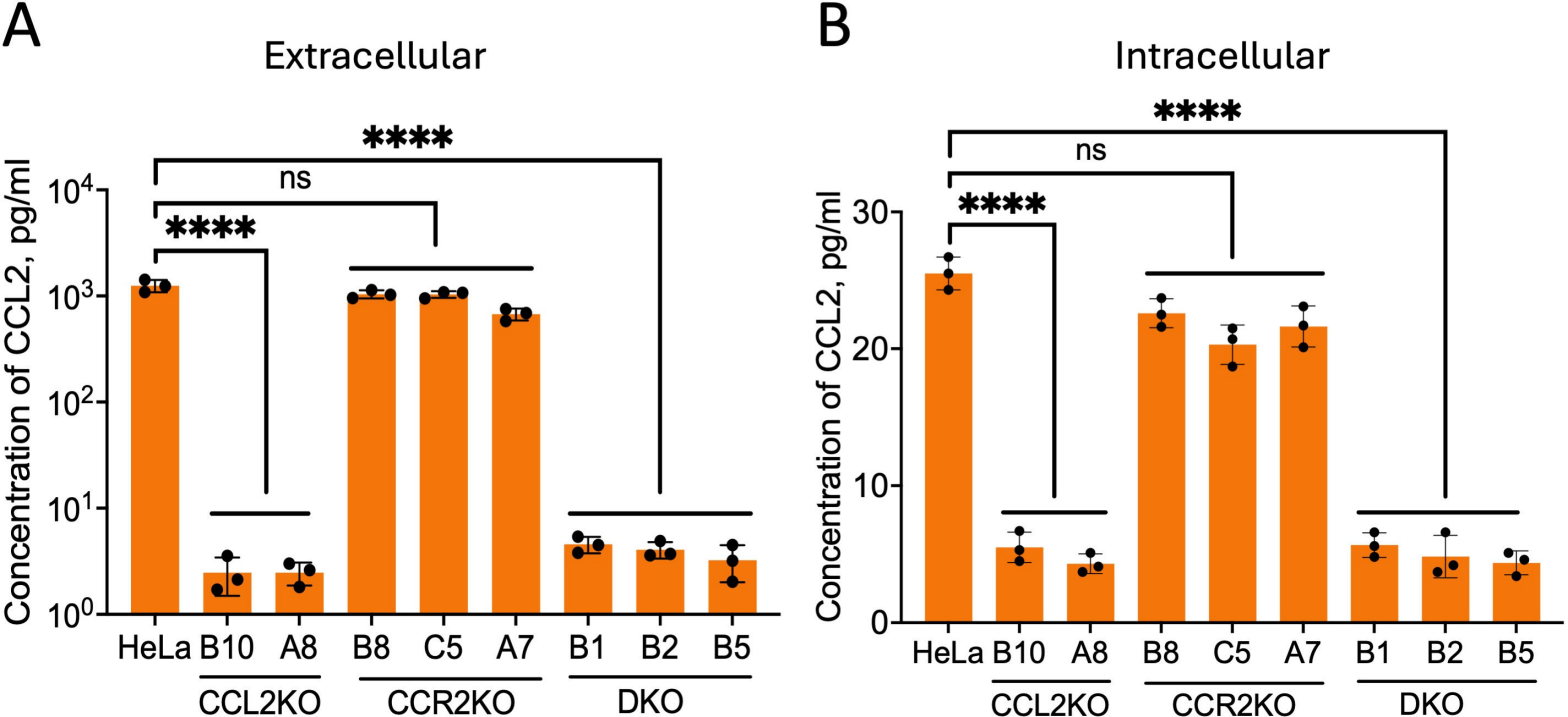
Verification of CCL2 production levels in parental HeLa cells and CCL2/CCR2KO cells. All cell lines, CCL2KO (B10 and A8), CCR2KO (B8, C5, and A7), and DKO cells (B1, B2, and B5) were seeded in 6-well plates at a density of 5 × 10^5^ cells. (**A**) Extracellular CCL2: 1 mL of media was collected after 24 h, clarified, and CCL2 levels were measured via Luminex assay. The picogram amount plotted corresponds to 50% of the total extracellular CCL2. (**B**) Intracellular CCL2: cell pellets from each culture were lysed as described in Materials and Methods, and CCL2 levels were measured. The picogram amount plotted corresponds to 10 times that of total extracellular CCL2. The experiment contained triplicates and was performed three independent times. The data represent mean ± SEM of three independent experiments (*n* = 3).****P < 0.0001.

**Fig 2:**
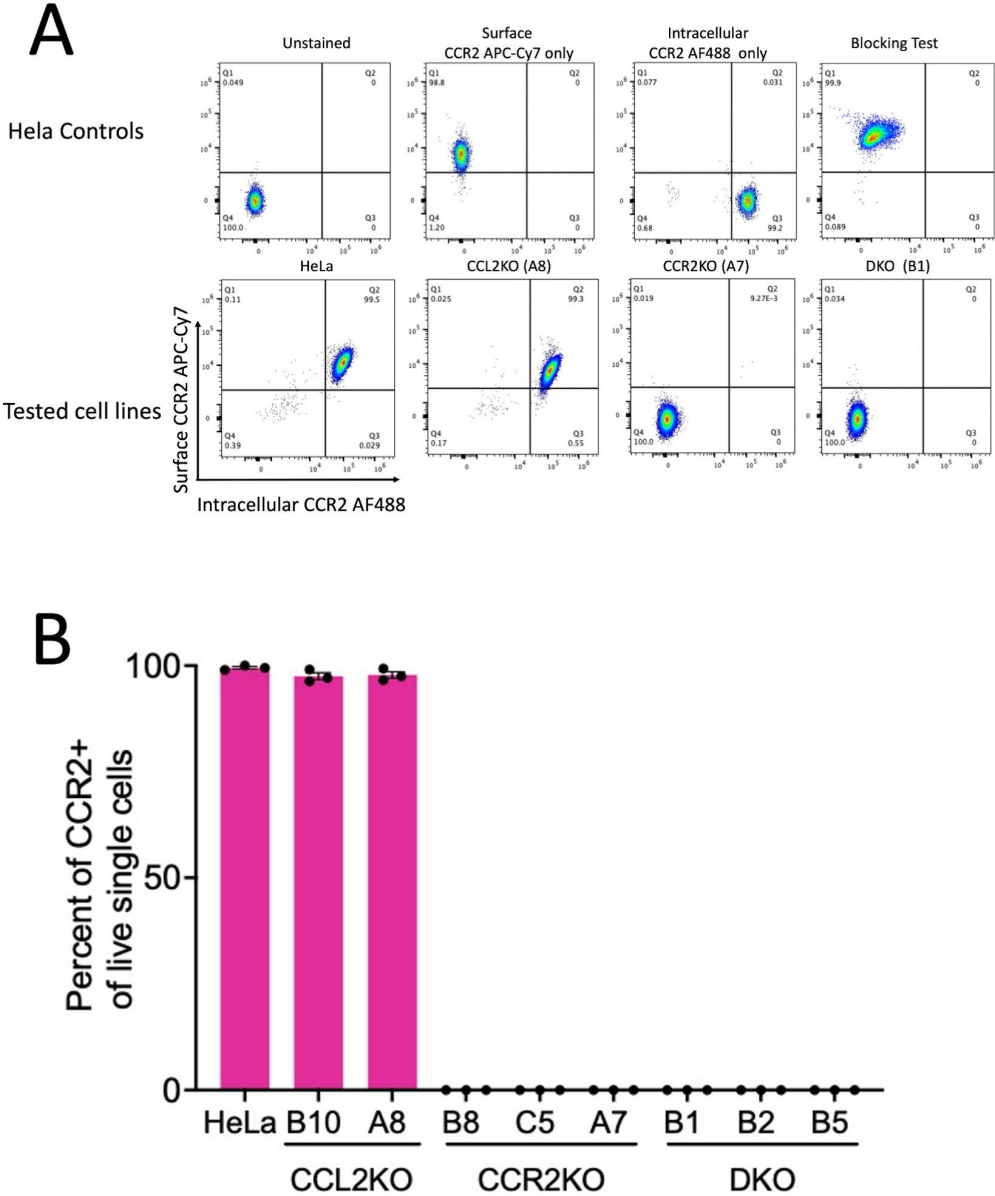
Detection and quantification of total CCR2 expression in parental HeLa cells and CCL2/CCR2KO cells via flow cytometry. (**A**) Gating strategy for CCR2 detection in parental HeLa, A8 CCL2KO , A7 CCR2KO, and B1 DKO cells. Representative dot plots show the expression of CCR2 on the cell surface (*y*-axis) and intracellularly (*x*-axis) across various conditions and cell lines. The top panel represents controls and validation, which includes unstained live cells, CCR2 extracellular APC Cy7-A, CCR2 intracellular AlexaFluor-488 A, and blocking test. The bottom panel represents the expression of the total surface-expressed and intracellular CCR2 shown for parental HeLa cells and one representative each of CCL2KO, CCR2KO, and DKO cells. Parental HeLa cells and A8 CCL2KO cells exhibited strong CCR2 expression, with the majority of cells (~99.5% and 99.3%, respectively) positive for both surface and intracellular CCR2 (Q2 quadrant). In contrast, A7 CCR2KO and B1 DKO, which lacked CCR2 expression, had >99.9% of cells in the double-negative quadrant (Q4), confirming receptor knockout. (**B**) Graph shows a plot of quantification of total CCR2 (surface and intracellular) for all cell lines as a percentage of total CCR2 expressed in parental HeLa cells. High levels of CCR2 expression were detected in parental HeLa and CCL2KO cells, whereas CCR2KO and DKO cells showed undetectable CCR2 expression, confirming successful gene knockout in those lines. The experiment was conducted three times. Data for panel A show representative examples, and panel B shows mean ± SEM of three independent experiments (*n* = 3).

To determine the effect of knockout, the levels of extracellular (secreted) and intracellular CCL2 were determined via Luminex assay for HeLa, CCL2KO (lines B10 and A8), CCR2KO (lines B8, C5, and A7), and DKO (lines B1, B2, and B5) cell lines. Parental HeLa cells and the three CCR2KO cell lines released robust levels of CCL2 into the medium (~2,000 pg/mL) (Fig. 1A) and the corresponding intracellular levels, in cell lysates, were at 2–2.5 pg/mL (Fig. 1B). In contrast, CCL2KO and DKO cell lines showed background levels of CCL2, which were at or near the detection limit. Next, we measured the cell surface and intracellular levels of CCR2 in parental and KO HeLa cell lines via flow cytometry. We first stained the cell surface-expressed CCR2 using APC-Cy7-labeled anti-CCR2 antibodies, washed off the excess antibodies, permeabilized cells, and then stained intracellular CCR2 with Alexa488-labeled anti-CCR2. Our results showed a robust level of expression of CCR2 in the parental HeLa cells and the CCL2KO lines B10 and A8. In contrast, CCR2 was not detectable in CCR2KO and DKO cells. Quantification of relative CCR2 levels (intracellular and surface expressed) in HeLa cells and in one representative cell line for each of the three KO cell lines, A8 CCL2KO , A7 CCR2KO , and DKO B1, is shown in Fig. 2A. The levels of surface and intracellular expression of CCR2 in the B10 CCL2KO were similar to those in parental HeLa cells or A8 CCL2KO cells, whereas the levels in the CCR2KO B8 and C5 lines and in DKO B2 and B5 were at baseline levels similar to DKO B1 cells, as shown in Fig. 2A (data not shown). Quantification of relative CCR2 levels in all nine cell lines is shown in Fig. 2B. Thus, our results confirm that the genetic deletion of CCL2 or the CCR2 genes leads to the absence of expression of these proteins in these cell lines.

### Effect of abrogation of CCL2 signaling on the mobilization of ALIX from the actin cytoskeleton

**Fig 3:**
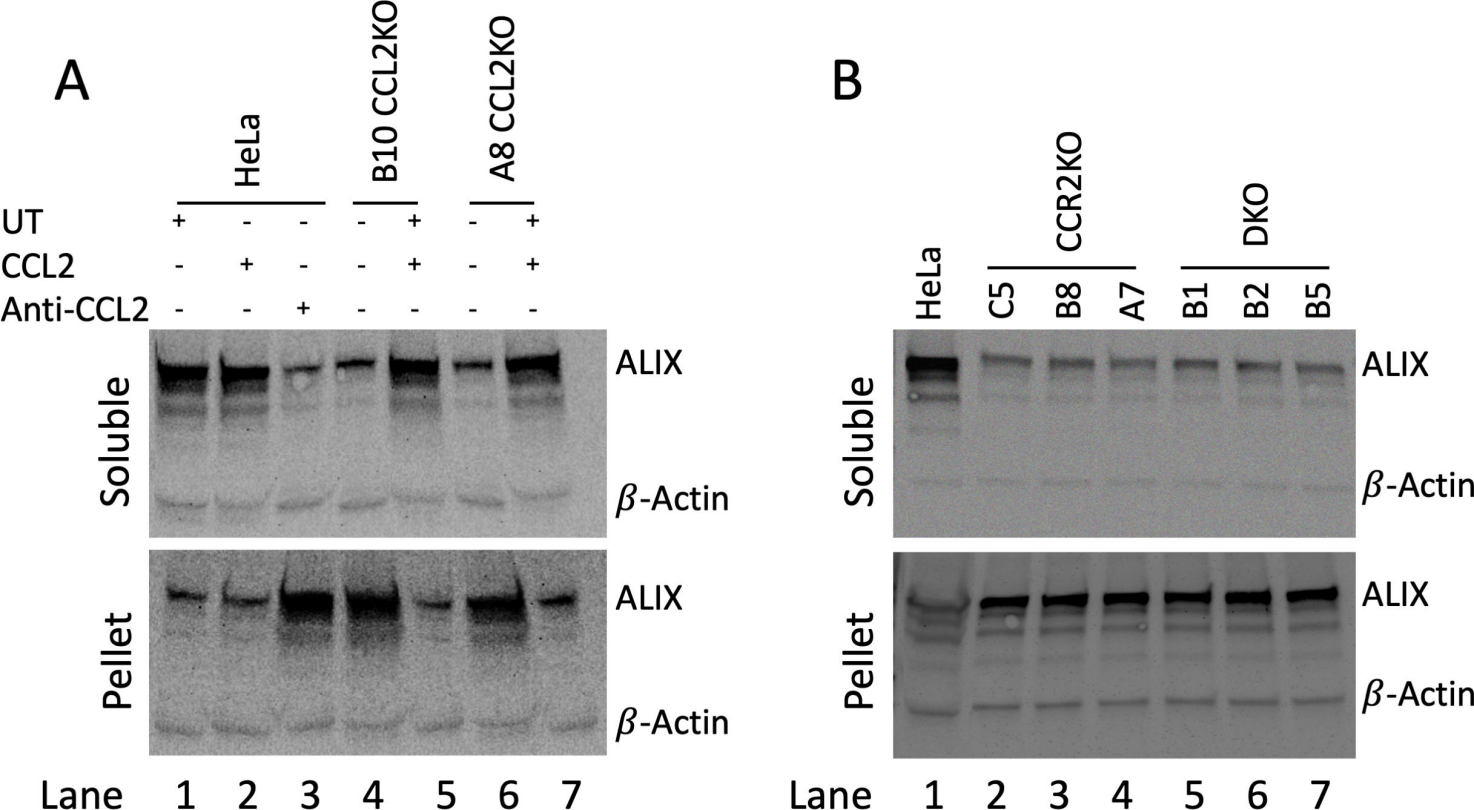
Soluble and insoluble ALIX in parental HeLa and the CCL2/CCR2KO cells. (**A**) Soluble (top panel) and insoluble (bottom panel) ALIX in parental HeLa and CCL2KO cells. It shows untreated, CCL2-treated, and anti-CCL2-treated parental HeLa cells, as well as untreated and CCL2-treated CCL2KO cells (A8 and B10).(**B**) Soluble (upper panel) and insoluble (pellet) ALIX in parental HeLa, CCR2KO, and DKO cells. It shows parental HeLa cells, CCR2KO cells (B8, A7, and C5), and DKO cells (B1, B2, and B5). The experiment was conducted three independent times.

We previously showed that CCL2 signaling mobilizes ALIX from F-actin cytoskeletal structures into the soluble cytosol, thereby enhancing HIV-1 budding and release. We also showed that blocking CCL2 signaling by the addition of neutralizing anti-CCL2 antibodies causes ALIX to be sequestered on F-actin, strongly inhibiting HIV-1 budding and release (2). Therefore, we next examined the ALIX protein levels and distribution in the soluble and F-actin cytoskeletal fractions of parental HeLa cells and all KO cell lines. In parental HeLa cells, most of the ALIX was observed in the soluble fraction both with and without CCL2 treatment. However, when cells were treated with anti-CCL2 antibodies, very little ALIX was recovered in the soluble fraction. We also observed that when most of the ALIX was in the soluble fraction, the insoluble fraction (P2 pellet) contained only residual amounts of ALIX (Fig. 3). The converse was also true when the insoluble fraction contained most of the ALIX (compare “Soluble” and “Pellet” panels of Fig. 3A, lanes 1–3). In contrast to parental HeLa cells, the CCL2KO cell lines, B10 and A8, showed only low levels of ALIX in the soluble fraction, but addition of CCL2 to these cultures increased the amount of ALIX in the soluble compartment (Fig. 3A, lanes 4–7). Similar to CCL2KO cells, the CCR2KO cells (C5, B8, and A7) and DKO cells (B1, B2, and B5) had low levels of soluble ALIX and relatively higher levels of insoluble ALIX (Fig. 3B, compare top and bottom panels). These results indicate that the knockout of CCL2, CCR2, or both genes in these cells enhances the association of ALIX with the F-actin cytoskeleton, and that treatment of CCL2KO cells with CCL2 reversed this phenotype.

**Fig 4:**
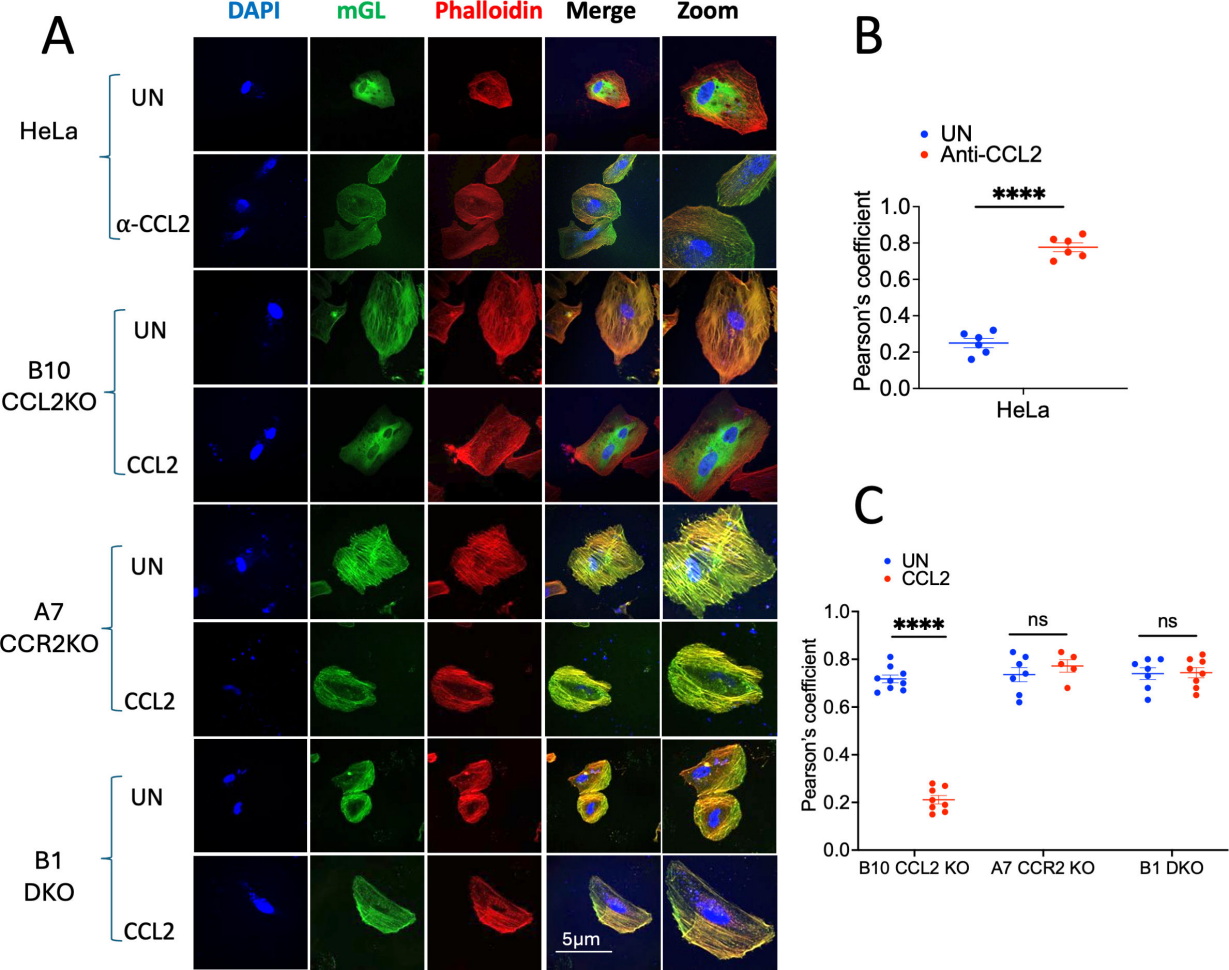
Colocalization of ALIX-mGreenlantern with F-actin under CCL2 signaling blockade and its reversal. (**A**) Confocal microscope images of HeLa cells (rows 1 and 2), B10 (CCL2KO) cells (rows 3 and 4), B8 (CCR2KO) cells (rows 5 and 6), and B1 (DKO) cells (rows 7 and 8) transfected with mGreenLantern. Sixteen hours later, cells were processed as described in Materials and Methods and imaged using a confocal microscope. The results show that ALIX-mGreenLantern (green channel) is organized into stress fiber-like structures in HeLa cells when treated with anti-CCL2 in B10 in the absence of CCL2 treatment and in B8 and B1 in the presence or absence of CCL2 treatment. On the other hand, ALIX-mGreenLantern is diffusely distributed in the cytosol when HeLa cells are untreated (UN) and when B10 is treated with CCL2. (**B**) Pearson’s colocalization coefficient was calculated to measure the degree of colocalization of ALIX-mGreenLantern with F-actin in untreated or anti-CCL2 (α-CCL2) treated HeLa cells. (**C**) Pearson’s colocalization coefficients were calculated in UN or CCL2-treated B10 CCL2KO, A7 CCR2KO, and B1 DKO. For each KO cell line, we used a minimum of five cells for quantitation (range +5–9). Scale bars for all images (except zoomed images) (5 µm) and for zoomed images (10 µm) are shown in the merged channel image or its zoomed version in the bottom row, respectively. ****P < 0.0001.

The above results, showing that CCL2 signaling blockade via CCL2 or CCR2 knockout leads to ALIX sequestration with F-actin, are in agreement with our previous findings obtained via the use of anti-CCL2 antibodies (2). In order to verify these biochemical results by a second approach, we developed a novel approach to detect ALIX-F-actin colocalization by employing a fluorescent protein-tagged ALIX. We first generated an ALIX-mGreenLantern fusion construct by combining ALIX and mGreenLantern via overlap polymerase chain reaction (PCR) (see Materials and Methods). We transfected the ALIX-mGreenLantern expression construct into parental HeLa cells and representative cell lines from each of the three classes of KO cell lines (CCL2KO, CCR2KO, and DKO), followed by confocal microscopy to detect F-actin and ALIX-mGreenLantern colocalization. Untreated parental HeLa cells, as expected, show a diffuse distribution of ALIX-mGreenLantern throughout the cytosol and distinct F-actin stress fibers without any ALIX-mGreenLantern associated (Fig. 4, first row from the top). Upon treatment with anti-CCL2, however, parental HeLa cells displayed colocalization of mGreenLantern and F-actin. This can be seen both in the green channel, which shows ALIX-mGreenLantern organized into stress fibers, and the merged green and red channels, which show the presence of yellow stress fibers and the absence of diffuse ALIX-mGreenLantern (Fig. 4, second row from the top). All three KO cell lines tested, namely, B10 (CCL2KO), A7 (CCR2KO), and B1 (DKO), showed ALIX-mGreenLantern colocalization with F-actin. Furthermore, CCL2 treatment of B10 showed reversal of the above phenotype, resulting in a diffuse distribution of ALIX-mGreenlantern in the cytosol. CCL2 treatment of A7 and B1, however, had no effect on the colocalization of ALIX-mGreenLantern and F-actin due to the fact that these cells lack the CCR2 receptor (Fig. 4). The results obtained by visual inspection of the ALIX (green) channel and the merged channels were verified by calculating the Pearson colocalization coefficients. These results, plotted in Fig. 4B and C, confirm the above conclusions. Collectively, the results of confocal microscopy are in agreement with the biochemical findings (Fig. 3).

### CCL2 and CCR2 KO cell lines are blocked at the level of HIV-1 virion production

**Fig 5:**
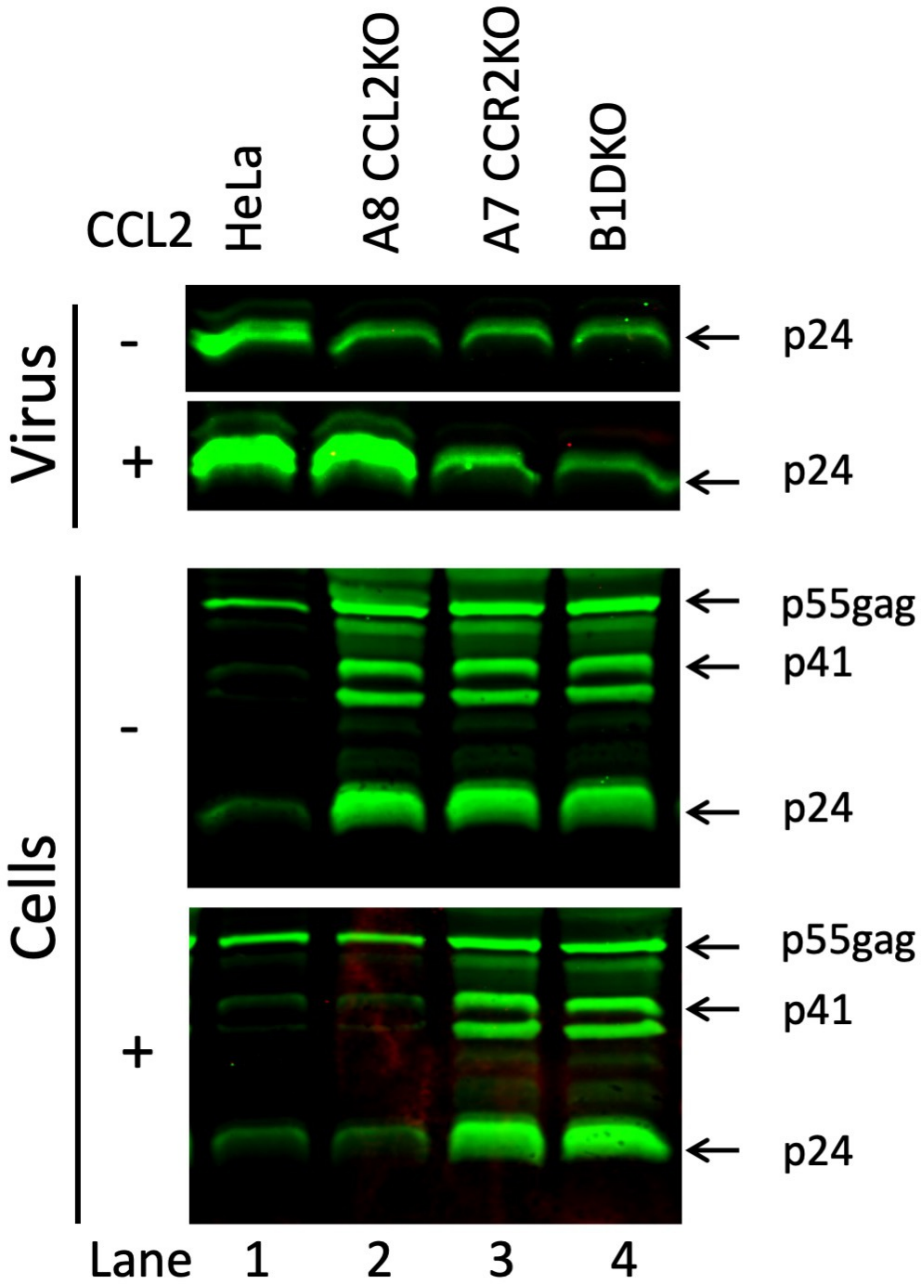
The effect of CCL2 signaling blockade on HIV-1 budding and release. Western blot shows that the HIV-1 virus production from parental HeLa cells and the KO cells A8 (CCL2KO), A7 (CCR2KO), and B1 (DKO) transfected with HIV-1_ADA_ is at the budding and release step. The top two rows show sucrose cushion-pelleted virus from the media, and the bottom two rows show cell lysates. All were probed with anti-p24 antibodies. The western blot is representative of three experiments.

In order to verify that our KO cell lines affect HIV-1 production at the budding and release stage similar to the effect we reported when HeLa cells or macrophages were treated with anti-CCL2 antibodies, we transfected the parental HeLa cells and representative cell lines from each of the three groups of KO cell lines—A8 (CCL2KO), A7 (CCR2KO), and B1 (DKO) with HIV-1_ADA_ molecular clone. While one set of samples was left untreated, a second set of cells was treated with CCL2. About 36 h post-transfection, the cell lysates and virion-containing supernatants were collected. The supernatants were pelleted through a sucrose cushion to obtain the virions. Both cell lysates and virions were subjected to western immunoblot analysis of both the virions and the cell lysate using an anti-p24 antibody. Our results show that, in the untreated samples, the parental HeLa cells produced the maximal amount of virus, which was further stimulated in the CCL2-treated sample (Fig. 5, lane 1, top two panels). Among the three KO cell lines, the production of virions was reduced compared to controls (Fig. 5, lane 1, top panel). However, the A8 (CCL2KO) cell line showed stimulation of virus production upon addition of CCL2, consistent with the fact that these cells retain CCR2 and thus the inhibition is reversible (Fig. 5, lane 2, top two panels). The two cell lines that lack CCR2—A7 (CCR2KO) and B1 (DKO)—however, showed no change in virus production (Fig. 5, lanes 3 and 4, top two panels).

The analysis of cell lysates was consistent with that of virions. When virus release was robust, the amount of p24 or p55 we detected in cell lysates was minimal (e.g., parental HeLa cells with or without CCL2 treatment and A8 CCL2KO cells treated with CCL2; Fig. 5, lanes 1 and 2, bottom two panels). On the other hand, when the virus production was inhibited, there were robust amounts of p24 and p55 observed in the cell lysates (A8 CCL2KO cells with no CCL2 added or in A7 CCR2KO or B1 DKO cells; Fig. 5, bottom two panels, lanes 3 and 4). These amounts did not change with CCL2 treatment of these cells (Fig. 5, bottom two panels). Thus, our results clearly demonstrate that the signaling blockade in these KO cell lines blocks HIV-1 virus production similar to when parental HeLa cells are treated with anti-CCL2 antibodies.

### CCL2 signaling blockade inhibits the production of wild-type HIV-1_ADA_ and HIV-1_ADA_^-PTAP^ but not HIV-1_ADA_^ΔLY^

**Fig 6:**
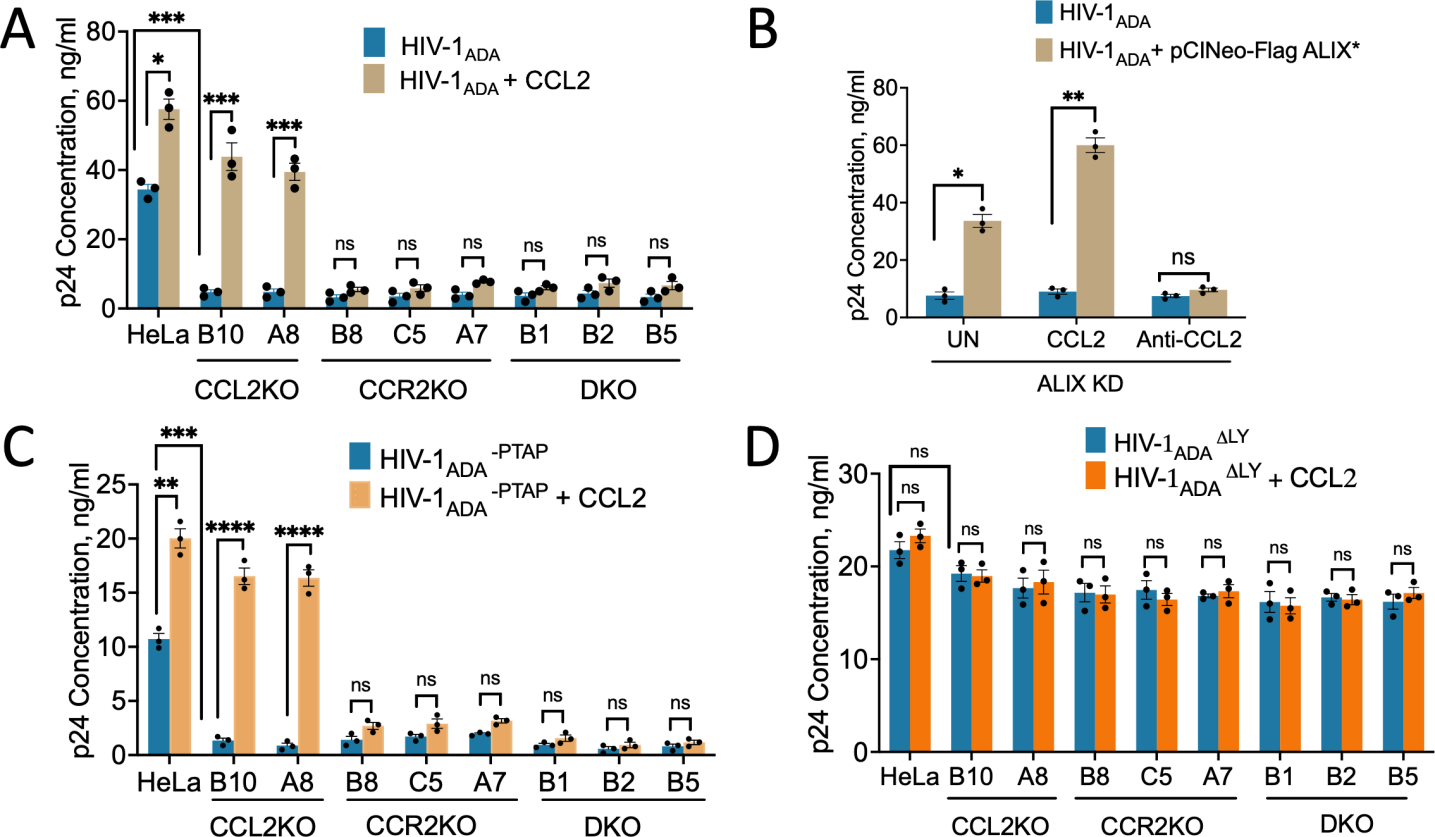
Effect of CCL2 signaling on the wild-type HIV-1_ADA_ and its two late domain mutant viruses. (**A**). Virus production was compared in parental HeLa cells and in each of the eight CCL2/CCR2KO cell lines after transfection with HIV-1_ADA_. In each cell line, virus production was measured with and without the addition of CCL2. (**B**) Inhibition of virus production in ALIX knockdown cells is similar to that observed in CCL2/CCR2KO cell lines. Virus production in ALIX KD cell line was measured in untreated, CCL2-treated, and anti-CCL2-treated conditions. (**C**) Virus production after transfection of parental HeLa cells and KO cells with HIV-1_ADA_^-PTAP^ with and without CCL2 treatment. (**D**) Virus production after transfection of parental HeLa cells and KO cells with HIV-1_ADA_Δ^LY^ and with and without CCL2 treatment. The data represent mean ± SEM of three independent experiments (*n* = 3). *P <0.05; ***P <0.001; ****P < 0.0001; and ns, not significant.

Next, we tested the effect of abrogating CCL2 signaling via CCL2KO and/or CCR2KO on the release of HIV-1_ADA_ virus and its L domain mutants. We transfected CCL2KO, CCR2KO, and DKO cells with an infectious molecular clone of HIV-1_ADA_ and measured p24 release via p24 ELISA after 24 h. Genetic deletion of CCL2, CCR2, or both significantly inhibited (7- to 12-fold) the production of HIV-1_ADA_ compared to parental HeLa cells (Fig. 6A). These results are comparable to the 8- to 10-fold inhibition we observed in HeLa cells or monocyte-derived macrophages using neutralizing anti-CCL2 antibodies (2). The addition of exogenous CCL2 in the medium led to an approximately 60% increase in virus production in HeLa cells. Interestingly, exogenous CCL2 addition also rescued virus production in the two CCL2KO cell lines, B10 and A8, to a level well above that of untreated HeLa cells (Fig. 6A). However, the CCR2KO and the DKO cell lines did not respond to CCL2 addition.

The impact of CCL2 or CCR2 knockout on HIV-1 production is expected to be due to the unavailability of ALIX for recruitment by the Gag-p6 late domain. If this hypothesis is correct, ALIX knockdown (KD) cells should have the same phenotype as CCL2KO or CCR2KO cells. In addition, the reintroduction of a shRNA-resistant ALIX (ALIX*) into ALIX KD cells should restore virus production to levels observed in the untreated parental HeLa cells. Therefore, we first created a HeLa cell line with a stable ALIX knockdown via lentiviral transduction (23) (Fig. S2). We then co-transfected HIV-1_ADA_ along with a shRNA-resistant ALIX (ALIX*) expression construct into the HeLa ALIX KD cell line, and the cells were either left untreated or treated with CCL2 or anti-CCL2. The transfection of HIV-1_ADA_ into the ALIX KD cell line resulted in severe inhibition of virus production, similar to CCL2KO or CCR2KO cells. Co-transfection of HIV-1_ADA_ with the ALIX* expression construct restored virus production to the level observed in parental HeLa cells. The addition of CCL2 or anti-CCL2 further stimulated HIV-1 production or inhibited it, respectively (Fig. 6B).

We next tested the impact of CCL2 or CCR2 KO on late events of HIV-1_ADA_^-PTAP^ mutant virus replication. We found that the absence of the PTAP motif resulted in a significant reduction of viral yields (approximately threefold) compared to the wild-type HIV-1_ADA_ in HeLa cells. However, as all of the PTAP^−^ mutant virus production occurs through the LYPX-ALIX pathway in HeLa cells, addition of CCL2 enhanced virus production by approximately twofold. In marked contrast, in the KO cell lines, the PTAP mutation resulted in a large reduction in viral production by ~5- to 17.5-fold when compared to parental HeLa cells. The reduction in virus yield is much more severe by ~17- to 57-fold, compared to parental HeLa cells transfected with the wild-type HIV-1_ADA_ DNA (Fig. 6A and C). This is due to the fact that while the PTAP^−^ mutation inhibits the PTAP-TSG101 pathway, the LYPX-ALIX pathway is disrupted in CCL2KO or CCR2KO cells due to sequestration of ALIX to the F-actin cytoskeleton. Thus, the combination of the PTAP^−^ mutation in the virus and the CCL2 signaling blockade in the KO cells together caused a robust inhibition of virus production. Addition of CCL2 stimulated viral production of HIV-1 ^-PTAP^ in parental HeLa cells and in CCL2KO cells (Fig. 6C). We note that the PTAP^−^ and ΔLY mutations specifically reduced virus production by two-thirds or one-third, suggesting that these mutations specifically inhibit the PTAP or the LY pathways.

The HIV-1_ADA_Δ^LY^ virus, which lacks the LY dipeptide but has the PTAP motif, showed roughly 30% inhibition in parental HeLa cells. This is consistent with our previous observation that the LYPX-ALIX pathway contributes approximately a third of the virus budding (2). The difference in the virus production for HIV-1_ADA_Δ^LY^ between parental HeLa cells and all the KO cells was not statistically significant. These data indicate that PTAP-mediated HIV-1 budding is not regulated by CCL2 signaling. It is worth noting that the deletion of the LY dipeptide in HIV-1 eliminated CCL2-responsiveness, as CCL2 addition did not affect ΔLY mutant viral release in either the parental HeLa cells or the KO cells (Fig. 6D). These results reinforce the fact that CCL2 responsiveness depends on the presence of the LYPX motif in the wild-type HIV-1_ADA_.

### SIV and EIAV budding and release are also affected by the abrogation of CCL2 signaling

In addition to HIV-1, several other retroviruses also use the LYPX late domain for budding—e.g., SIV, EIAV, RSV, and MuLV. We selected two of these viruses, SIV and EIAV, for further evaluation. SIV, like HIV-1, uses both PTAP and LYPX, whereas EIAV solely uses the LYPX motif. We examined the impact of the loss of CCL2 signaling on the budding and release of these two lentiviruses using constructs that encode the corresponding Gag proteins to facilitate the release of virus-like particles (VLPs). Wild-type and the late domain mutant Gag constructs were transfected into parental HeLa cells or the A8 CCL2KO cells. As both the PTAP and LYPX pathways are functional in HeLa cells, one can switch off virus production from specific pathways by mutating the respective late domains in the virus. In the A8 cells, virus production occurred only via the PTAP-TSG101 pathway, but the LYPX-ALIX pathway can be activated simply by adding CCL2 to the medium.

**Fig 7:**
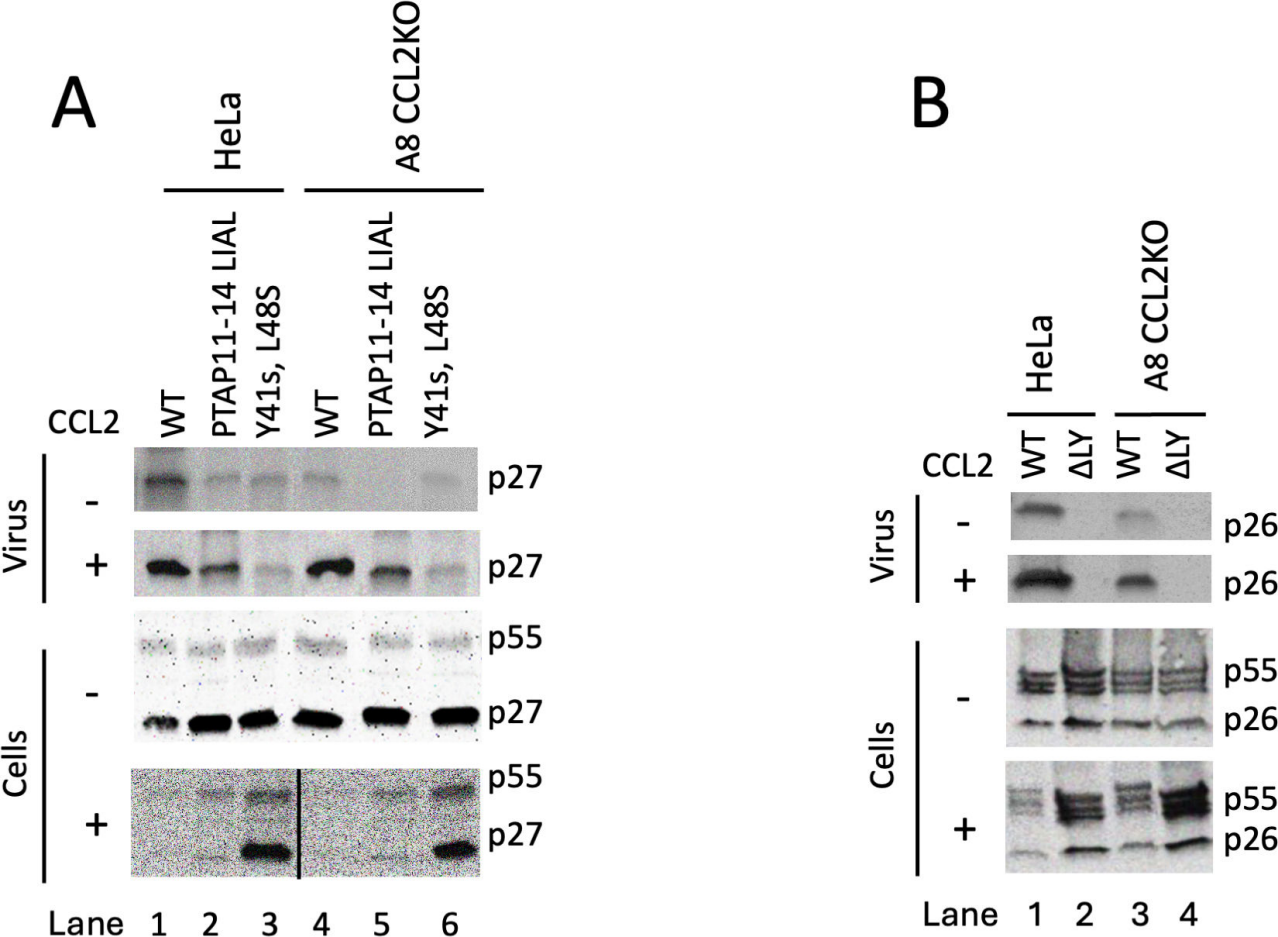
Examining the role of the CCL2-ALIX axis on the budding and release of SIV and EIAV. (**A**) Western blot shows the effect of CCL2 signaling blockade on the budding of SIV (WT) and its late domain mutants PTAP11-14 LIAL and Y41s, L48S, respectively, in parental HeLa cells and A8 CCL2KO . The top two panels show p27 in the virus pellet without and with CCL2 addition, and the bottom two panels show p55 and p27 in cell lysates without and with CCL2 addition. (**B**) Western blot shows the effect of CCL2 signaling blockade on the budding of EIAV (WT) and its mutant △LY in parental HeLa cells and A8 CCL2KO . Western blots in both panels A and B are representative of a minimum of three experiments each.

We transfected pSIV480 (WT) and its PTAP− and ΔLY mutants—pSIV481 (PTAP11-14 LIAL) and pSIV482 (Y41s, L48S)—into each of the above two cell lines. VLPs, which were pelleted through a 20% sucrose cushion, and the cell lysates containing Gag were both subjected to immunoblot analysis to detect virion-associated and cell-associated viral capsid protein (CA) levels (Fig. 7A). Transfection of the SIV-WT VLP construct into parental HeLa cells showed a robust VLP production compared to A8 CCL2KO cells, and both could be stimulated to higher levels by the addition of CCL2 to the cultures (Fig. 7A, top two panels, lanes 1 and 4). Both the PTAP^−^ and ΔLY mutations partially inhibited SIV VLP release in untreated parental HeLa cells, as the cells retained one of the two redundant pathways of virus budding. Reductions in virion-associated CA (p27) (Fig. 7A, compare lanes 2 and 3 with lane 1, top panel) were associated with a slight increase in cell-associated CA (p27) protein levels for both mutants (Fig. 7A, compare lanes 2 and 3 with lane 1, bottom panel). CCL2 treatment also increased VLP release for both wild type and the PTAP mutant, but not for the ΔLY mutant (Fig. 7A, lanes 1, 2, and 3), confirming the requirement for an intact LYPX for CCL2-mediated increases in virus production. In the A8 CCL2KO cell line, both wild-type virus and ΔLY mutant virus showed a partial inhibition of virus production, suggesting that only the PTAP-TSG101 pathway is functional in both viruses, while the PTAP mutant showed no detectable virus due to the fact that both the PTAP and LYPX pathways are defective in this specific virus mutant-KO cell line combination (Fig. 7A, lanes 4–6). In the KO cell line, the addition of CCL2 fully restored the production of wild-type virus, partially restored the PTAP^−^ mutant, and did not affect the LY mutant virus. For the ΔLY mutant in particular, the cell-associated p27 was most robust in both the parental HeLa and A8 cell lines, in agreement with very little virus produced with or without CCL2 addition.

While many retroviruses have more than one late domain, EIAV contains a single late domain motif, the LYPX. Thus, ESCRT III recruitment occurs only through the LYPX-ALIX pathway without the participation of redundant budding pathways (24, 25), which makes EIAV an attractive model for investigating the role of the CCL2-ALIX axis in viral budding. Figure 7B shows the results of EIAV (pEV53) and its mutant (pEV53 ΔLY) in parental HeLa cells or A8 CCL2KO cells with and without the addition of CCL2. As expected, the level of wild-type EIAV (pEV53) VLP production in parental HeLa cells is stimulated by the addition of CCL2, as indicated by the increase in virion-associated CA (p26) (Fig. 7B, virus western blot, compare lane 1, top two panels). EIAV (pEV53) virus production and release were significantly reduced in A8 CCL2KO cells, compared to parental HeLa cells, which exhibited a relative increase in cell-associated CA (p26) compared to parental HeLa cells (Fig. 7B, cellular western blot, compare lanes 1 and 3). Addition of CCL2 resulted in the stimulation of EIAV virion release in both parental HeLa cells and A8 CCL2KO cells (Fig. 7B, top panels, lanes 2 and 4). Stimulation of viral particle release in cultured media resulted in a significant reduction of cell-associated CA (p26) in both parental HeLa and A8 CCL2KO cells (Fig. 7B, cellular western blot, lanes 2 and 4, bottom panels). On the other hand, the ΔLY mutation leads to the absence of detectable virion-associated CA (p26) in both Parental HeLa cells and A8 CCL2KO cells (Fig. 7B, virion western blot, lanes 2 and 4, top panel). In addition, the ΔLY mutant construct did not respond to CCL2, showing no detectable virus-associated CA (p26) in both cell lines and a marked increase of cellular-associated CA (p26) (Fig. 7B, virion western blot, lanes 2 and 4, compare all panels).

### Blockade of CCL2 signaling does not affect the replication of HSV-1 or DENV

**Fig 8:**
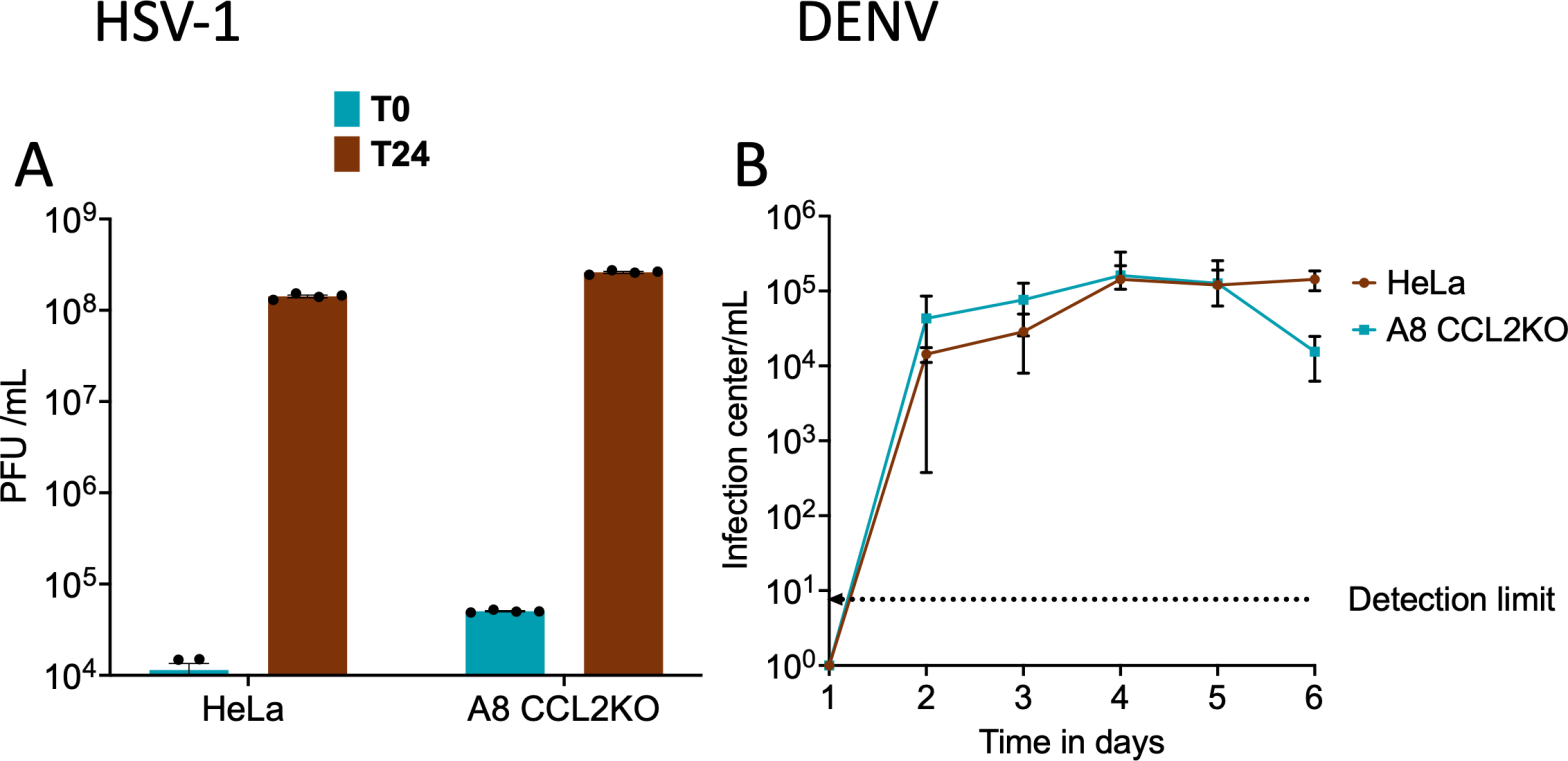
CCL2-ALIX axis does not operate in the replication of HSV-1 and DENV. (**A**) Replication of HSV-1 strain GS6807 on parental HeLa cells and A8 CCL2KO cells. After infection (MOI, 10), cells were collected either immediately (T0) or after 24 h (T24). Titration of whole-cell extracts via plating onto pre-established Vero cell monolayers was used to measure PFU. (**B**) DENV2 growth on parental HeLa and A8 CCL2KO cells. Indicated cell lines were infected with DENV2 16681 at low MOI. Culture media were collected at 1-day intervals and titrated on Vero cells using an infectious center assay. The data represent the mean ± SEM of three independent experiments (*n* = 3).

It is known that cytoplasmic envelopment of HSV-1 can be inhibited by the expression of a dominant negative form of the terminal ESCRT ATPase vacuolar protein sorting 4 (Vps4) (26). However, the mechanism by which ESCRT-III and Vps4 are recruited by herpes viruses is not well understood (27). It has also been reported that depletion of ALIX in HSV-1-infected parental HeLa cells results in anapproximately fourfold increase in the numbers of partially enveloped HSV-1 capsids at the inner nuclear membrane, consistent with a role for ALIX in primary envelopment (28). Since parental HeLa cells support HSV-1 replication, we employed our CCL2KO cell system to investigate whether the unavailability of ALIX affects the replication of HSV-1. We infected parental HeLa cells and A8 CCL2KO with HSV-1 at a MOI of 10. After 1 h of infection, we inactivated the unpenetrated virus, and the infected cells were either lysed immediately at this point (T0) or after an additional 24 h (T24), and the total infectious yields of HSV-1 were determined by titration on Vero cells. Our results (Fig. 8A) show that the unavailability of ALIX in A8 CCL2KO cells did not affect HSV-1 virus production.

Another interesting example is DENV. In previous studies, the replication of DENV and Japanese Encephalitis Virus (JEV) was reported to be robustly affected by siRNA-mediated depletion of TSG101 or charged multivesicular body proteins CHMP3 and CHMP4 (ESCRT III), suggesting ESCRT-mediated budding and release are involved in DENV replication. Interestingly, DENV replication was unaffected by depletion of ALIX in another study (29). This was surprising as the related flaviviruses, such as yellow fever virus (30) and tick-borne encephalitis virus (31), are known to require ALIX for replication. We selected DENV as an example and determined the growth kinetics of DENV in parental HeLa cells and A8 CCL2KO cells. Cells were infected with DENV2 16681 at a MOI of 0.01 infectious centers per cell, and the media were harvested every 24 h post-infection over several days and titrated via an infectious center assay. As shown in Fig. 8B, the CCL2 signaling blockade had no detectable effect on DENV production.

### HAZV replication is affected by the abrogation of CCL2 signaling

**Fig 9:**
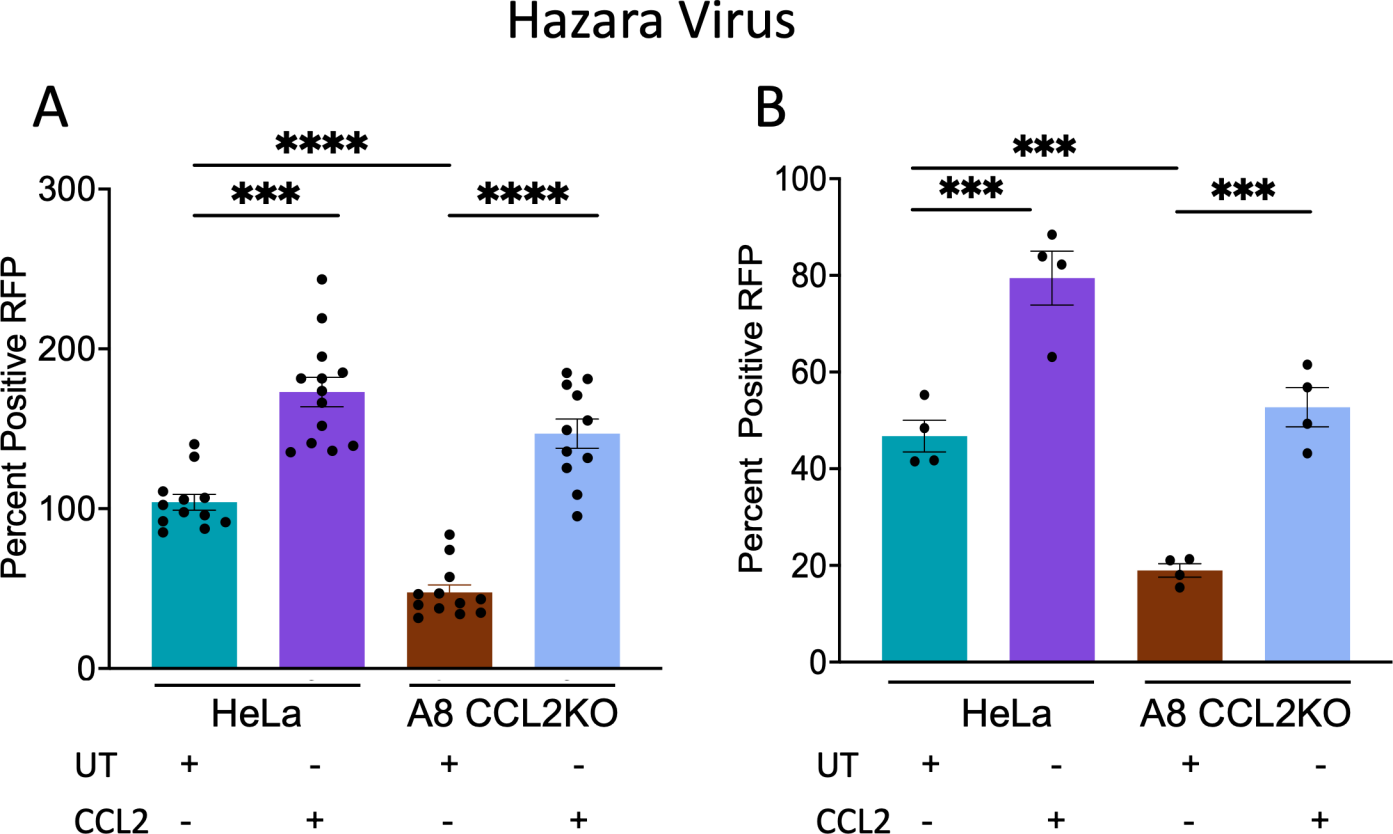
CCL2-ALIX axis operates in the replication of HAZV detected by immunofluorescence assay. (**A**) The release and production of HAZV in infected SW-13 cells infected with HAZV-containing supernatants harvested from parental HeLa cells (untreated and treated with CCL2, respectively), A8 CCL2KO cells (untreated and treated with CCL2, respectively) at a dilution of 1:800. (**B**) The production of HAZV in infected parental HeLa cells and A8 CCL2KO , with HAZV-containing supernatants harvested from parental HeLa cells at a dilution of 1:400. The data represent mean ± SEM of three independent experiments (*n* = 4). ***P <0.001; and ****P <0.0001.

HAZV and Crimean-Congo hemorrhagic fever virus (CCHFV) are members of the family Nairoviridae in the class *Bunyaviricetes* of enveloped viruses with segmented negative-strand RNA genomes. HAZV, avirulent in humans, is frequently employed as a surrogate for the highly virulent CCHFV (32). There are no prior studies implicating a role for ALIX or ESCRT components in HAZV or CCHFV budding and release, or indeed, in the budding and release of any member of *Bunyaviricetes*. Nevertheless, ALIX and ESCRT proteins have been shown to play a role in CCHFV entry through multivesicular bodies (33). These findings led us to investigate the role of CCL2 depletion on HAZV replication and release. To evaluate the role of CCL2 in HAZV replication, infectious virus particles were produced in parental HeLa cells or A8 CCL2KO (with and without CCL2 treatment), and the viral titers were evaluated in SW-13 cells. As shown in Fig. 9A, viral production was reduced by ~2.5-fold when the virus originated from A8 CCL2KO cells, compared to the virus derived from parental HeLa cells. Treatment with CCL2 led to an approximately twofold increase in viral production relative to untreated parental HeLa cells. Similarly, CCL2 treatment of A8 CCL2KO cells resulted in an approximately threefold increase in viral release compared to untreated A8 CCL2KO cells. As our assay involved the use of an additional cell line (SW-13) to measure the viral titer, we streamlined our assay by infecting parental HeLa cells or its A8 CCL2KO derivative and directly measuring virus production on the parental HeLa and A8 cells using an indirect immunofluorescence assay. Parental HeLa and A8 CCL2KO cells were infected with or without CCL2 treatment. Figure 9B shows ~2.6-fold inhibition of virus replication in A8 CCL2KO cells compared to parental HeLa cells. Treatment of both parental HeLa and A8 cells with CCL2 increased virus production by ~1.7- and 3-fold, respectively, compared to untreated cells. These findings indicated that the CCL2/ALIX signaling axis plays a role in HAZV replication.

## DISCUSSION

In this study, we have generated knockout cell lines, where CCL2, CCR2, or both have been genetically ablated, which could serve as novel tools for studying the role of CCL2 signaling in mobilizing ALIX and enhancing virus budding and release. We examined the effect of CCL2 signaling blockade on the mobilization of ALIX from the F-actin cytoskeleton to the cytosol and found that the loss of CCL2 or its receptor CCR2 in KO cells caused ALIX to associate with the F-actin cytoskeleton with significantly reduced levels in the cytosol. Conversely, the addition of exogenous CCL2 to CCL2KO cells led to the mobilization of ALIX from the F-actin cytoskeleton to the cytosol (Fig. 3A and 4), showing that the signaling defect is reversible. These findings were from both a biochemical analysis of F-actin cytoskeleton extracts and soluble fractions, as well as by confocal microscopy. Using our KO HeLa cell lines, we also tested the effect of CCL2 signaling blockade on the production of HIV-1_ADA_. First, by examining sucrose cushion-pelleted virus, we showed that the defect in replication was at the level of virion production (Fig. 5). Second, we compared the production of wild-type HIV-1_ADA_ and its two late domain mutant viruses—HIV-1_ADA_^-PTAP^ and HIV-1_ADA_Δ^LY^ in both the parental HeLa and all the KO cell lines to verify that the mechanism of CCL2-mediated HIV-1 budding was mediated by the LYPX and not the PTAP motif (Fig. 6). Wild-type HIV-1_ADA_ was produced at robust levels in parental HeLa cells and severely inhibited in all the CCL2KO and CCR2KO cell lines. In parental HeLa cells and CCL2KO cells, CCL2 addition enhanced HIV-1_ADA_ virus production to levels much higher than those observed in untreated parental HeLa cells. HIV-1_ADA_Δ^LY^ mutant virus displayed a degree of inhibition that resulted in roughly a third of the level of wild-type HIV-1_ADA_ virus production, consistent with the fact that the LYPX-ALIX pathway is a minor pathway of virus budding and release (34, 35). Importantly, the absence of LY dipeptide in this virus makes it unresponsive to CCL2 levels even in parental HeLa cells. In CCL2KO and CCR2KO cells, transfection of HIV-1_ADA_Δ^LY^ also yielded a similar level of inhibition. In other words, the methodology of blocking the LYPX-ALIX pathway, i.e., via LY deletion or CCL2/CCR2 ablation in the producer cells, does not appear to matter. Due to the LYPX-ALIX pathway being ablated via a late domain mutation, even in the +CCL2 controls, which make ALIX more available, there is no stimulation of virus production in all cell lines, including the parental HeLa cells (Fig. 6D).

Mutation of the PTAP motif reduced virus production by about two-thirds in parental HeLa cells, consistent with the fact that the PTAP-TSG101 pathway is the major pathway for HIV-1 budding and release. The inhibition in CCL2KO and CCR2KO cells was severe due to two distinct effects. First, the PTAP mutation inactivates the PTAP-TSG101 pathway, making the virus solely reliant on the LYPX-ALIX pathway. Second, the LYPX-ALIX pathway is rendered inactive in CCL2KO and CCR2KO cells due to the CCL2-signaling blockade and sequestration of ALIX to the F-actin cytoskeleton. However, in the B10 and A8 CCL2KO cell lines, PTAP^−^ virus production could be restored significantly by the addition of exogenous CCL2 (Fig. 6C). We note that the PTAP^−^ and ΔLY mutations specifically reduced virus production by two-thirds or one-third, respectively, suggesting that these mutations are specifically inhibiting the PTAP or the LYPX pathways. We also note that the remaining virus production in a PTAP^−^ virus can be further inhibited by CCL2 signaling blockade. Interestingly, however, when transfected into CCL2KO cells, the wild-type virus is inhibited strongly (~10-fold) even though presumably only the LYPX-ALIX pathway is being blocked. We do not know the basis of this strong, near-complete inhibition, which should be mediated solely via the LYPX-ALIX pathway. Considering that ALIX’s chief role is to recruit ESCRT III proteins, it is likely that the CCL2 signaling blockade inhibits common mediators of both LYPX-ALIX and PTAP-TSG101 release pathways.

Several other retroviruses use the LYPX-ALIX pathway, including SIV, EIAV, and MuLV. To examine the potential wider importance of the CCL2-ALIX axis, we examined SIV and EIAV. These two viruses are also known to induce CCL2 upon infection (36, 37). We examined both ALIX dependence and responsiveness to CCL2 levels for both of these viruses. SIV is a close relative of HIV-1 and uses both TSG101-binding PTAP and ALIX-binding LYPX domains for virus budding and release. Use of combinations of (i) late domain mutations; (ii) CCL2KO cell lines; and (iii) rescue of the virus LYPX-ALIX pathway by CCL2 addition allowed us to show the following. First, like HIV-1, SIV also needs the LYPX motif for enhanced virus production when CCL2 is added. Second, CCL2 signaling blockade in A8 CCL2KO cells does not affect budding of mutant viruses that exclusively use the PTAP-TSG101 pathway, showing the CCL2 effect is mediated only by the LYPX motif. Third, CCL2 signaling blockade can ablate the LYPX-ALIX pathway without the need to either mutate LYPX or silence ALIX. Our results are in agreement with a previous study by Zhai and colleagues (18), who reported that the PTAP and YPXL late domains in Gag p6 protein of SIVmac251 are crucial for virus release and infectivity. Mutations in these domains inhibit virus release, leading to the accumulation of Gag processing intermediates. Overexpression of ALIX stimulates SIVmac251 release, requiring YPXL late domain interaction. The same combination of conditions listed above was helpful to demonstrate that EIAV budding and release are also influenced by CCL2 signaling. Due to the presence of a single late domain, the results are uncomplicated and straightforward. CCL2 signaling blockade in A8 CCL2KO cells led to a significant reduction in EIAV (WT) virion release, whereas the addition of CCL2 stimulated the release of viral particles in both parental HeLa cells and A8 CCL2KO cells. However, the LY mutation in the EIAV Gag severely reduced viral production.

While the retroviruses we tested are proven to use the LYPX-ALIX pathway, the literature on the use of the LYPX-ALIX pathway for some other enveloped DNA and RNA viruses is either controversial or only beginning to be addressed. For HSV-1, even though LYPX and P(T/S)AP late domain-like consensus motifs have been identified in some of its structural proteins, the expression of dominant negative versions of ALIX or TSG101, and silencing of ALIX and TSG101 singly or together, had no impact on virus production (38). Nevertheless, a subsequent study reported that depletion of ALIX in HSV-1-infected parental HeLa cells results in an approximately fourfold increase in the numbers of partially enveloped HSV-1 capsids at the inner nuclear membrane, consistent with a role for ALIX in primary envelopment (28). However, the same study concluded that ALIX depletion had no effect on the number of HSV-1 capsids reaching the cytoplasm, nor the final yields of infectious HSV-1 virions. The ability of our CCL2KO cell line, which severely restricts the availability of ALIX in the soluble cytosolic pool, provides yet another robust approach to test if ALIX is involved in HSV-1 replication. Our results clearly show that CCL2 signaling blockade has no observable effect on the replication of HSV-1. Thus, our results are in agreement with the conclusions of Pawliczek and Crump (38) that ALIX is not required for HSV-1 primary envelopment at the inner nuclear membrane or secondary envelopment in the cytoplasm (27). These data are also consistent with the finding that HSV-1 does not require many of the early components of the ESCRT apparatus for primary or secondary envelopment, since the Bro1 domain proteins HD-PTP and Brox, and the ESCRT II component EAP20/VPS25, are also all individually dispensable for HSV-1 replication (38, 39).

Previous efforts using either siRNA targeting ALIX or dominant negative ALIX forms showed that ALIX is required for replication of orthoflaviviruses such as tick-borne encephalitis virus (31) or yellow fever virus (30). Interestingly, siRNA knockdown of ALIX had no effect on DENV or JEV replication (29). In the same study, siRNA targeting specific ESCRT proteins such as TSG101 or combinations of CHMP2/3 or CHMP4 dramatically decreased the release of DENV and JEV (29). The absence of a requirement for ALIX is quite surprising, as all four viruses are members of the same genus. Our studies investigating the contribution of ALIX to DENV replication in CCL2KO HeLa cells confirm these previous findings and provide further support to the conclusion that DENV does not utilize the ALIX pathway for its budding and release.

HAZV has been utilized as a surrogate system for the CCHFV, a member of the same viral family (Nairoviridae) (32). Recent studies have demonstrated the role of the ESCRT pathway in both CCHFV release and entry. ALIX is thought to contribute to CCHFV release, as shown by a potential inhibitor of the ALIX-YxxL motif (40). However, no YxxL-like motif has been identified in CCHFV or HAZV, and neither ALIX knockdown nor dominant negative ALIX has been shown to inhibit CCHFV or HAZV release thus far. However, silencing of TSG101, CHMP3, VPS4B, or ALIX—components of the ESCRT pathway that control MVB biogenesis—has been shown to impair the infection and entry of CCHFV, indicating that ESCRT plays a role in CCHFV entry (33). These findings encouraged us to examine the role of CCL2 signaling blockade on the replication of the HAZV. Our initial experimental setup required us to use a second set of cells, the SW13 cells, to titrate the infectious virus produced (Fig. 9A). As this second round of infection and virus release steps may be confounding due to two sets of entry and release involved, we repeated the experiment in HeLa cells and A8 KO cells and directly measured the infectious centers on these cells. Both experiments showed that the CCL2 signaling blockade inhibited virus replication. Due to limitations with reagents (e.g., antibodies) to directly measure virus production, we were unable to measure virus production separately from entry, and hence at this point, it is unclear if the CCL2-ALIX axis is required for entry, budding, or both. Nevertheless, the fact that the addition of CCL2 increased replication compared to untreated A8 CCL2KO cells confirms the importance of ALIX for HAZV replication (Fig. 9B).

## MATERIALS AND METHODS

### Cells

HeLa cells were obtained from ATCC (CRM-CCL-2). HeLa cells and their gene knockout derivatives were grown in DMEM (Gibco) supplemented with 10% fetal bovine serum (FBS) and 1% penicillin-streptomycin.

### Viruses

The macrophage-tropic HIV-1^ADA^ molecular clone and its mutant derivatives HIV-1_ADA_Δ^LY^ and HIV-1_ADA_^-PTAP^ were described previously (2). Molecular clones of SIV (pAd-SIV3+ [wt]) and its mutants (pAd-SIV3+[PTAP 11-14LIAL] and pAd-SIV3+[Y41S, L48S]) were as reported previously (18). Molecular clone of EIAV (pEV53-WISP99-54) was reported earlier (25). To generate the ∆LY mutant, we deleted the hexanucleotide GTCTAC from 1,369 to 1,374 bp in EIAV Gag p9 using Q5 Site-Directed Mutagenesis Kit (New England Biolabs). The presence of desired mutations and absence of undesired mutations were confirmed via nanopore and Sanger sequencing.

HSV-1 strain GS6807, derived from a self-excising strain F infectious clone, is wild type other than encoding a UL25-mCherry capsid fusion (41) and has been previously described (27, 39). HSV-1 stocks were grown and titrated on Vero cells grown in DMEM (Gibco Laboratories) supplemented with 10% newborn calf serum (Peak Serum, Inc.) as previously described (42).

### Generation of CRISPR/Cas9 knockout HeLa cell lines

Polyclonal CCL2KO, CCR2BKO, and CCL2KO/CCR2BKO (DKO) HeLa cell lines were generated using CRISPR-Cas9 technology (43). Two human CCL2 gRNA target sequences (CCL2 gRNA EX1 68/54: 5′-CAGCCACCTTCATTCCCCAA GGG-3′; CCL2 gRNA Ex2 62/68: 5′-CTGCACTGAGATCTTCCTAT TGG-3′) and two human CCR2B gRNA target sequences (CCR2B gRNA 32/92: 5′-GACTTCTTCACCGCTCTCGT TGG-3′; CCR2B gRNA 60/70:5′-TATCACATCGGTTATTTTGG CGG-3′) were designed using Benchling (https://www.benchling.com/). The CRISPR/Cas9 all-in-one plasmids encoding gRNAs, Cas9 nuclease, and puromycin selection marker were generated by SLiCE cloning strategy (44). HeLa cells were transfected using Lipofectamine 2000 with combinations of the CCL2 and/or CCR2B gRNA CRISPR/Cas9 all-in-one plasmids. Cells were cultured for 24 h and selected with 2 µg/mL puromycin for 48 h to enrich for transfected cells. Following single-cell cloning, regions surrounding the target site(s) for each gene were PCR amplified from the genomic DNA of transfected HeLa cells, cloned into a TA cloning vector, and 12 clones from each cell clone were sequenced via Sanger sequencing. Since HeLa cells are hypertriploid (45), we identified at least three alleles for each HeLa cell clone, with each allele displaying a distinct set of indels (Fig. S1).

### Measurement of CCL2 by Luminex assay

CCL2 in the media (extracellular) and in the cell lysates (intracellular) of all eight KO cell lines and the parental HeLa cell line was quantified via Luminex assay. Briefly, cells were seeded in 6-well plates at a density of 5 × 10^5^ cells/well, and 1 mL of cultured media was collected after 24 h. CCL2 in 25 µL each of the supernatants was measured by Luminex (Millipore, Billerica, MA, USA), using a HCYTA-60K-13 kit (Millipore Sigma). Data were acquired on a Luminex Magpix XMAP Multiplex Reader (Luminex Technologies) and analyzed using Belysa Immunoassay Curve Fitting Software (Millipore Sigma). Three independent experiments were performed.

### Measurement of CCR2 using flow cytometry

HeLa cells (parental and KO cells) were stained with Live/Dead dye, FC block, and surface markers, fixed and permeabilized using eBioscience Fixation/Permeabilization Kit according to the manufacturer’s protocol, and subsequently subjected to intracellular staining. The main reagents used for flow cytometry are viability—LIVE/DEAD Fixable Violet, FC block—Human TruStain FcX, surface—CCR2 APC-Cy7 (clone K036C2), and intracellular (Cyto)—CCR2 Alexa Fluor 488 (clone K036C2). All reagents were purchased from BioLegend or Thermo Fisher. FACS analyses were carried out using the Cytek Aurora Analyzer. A sequential blocking test was done with Surface CCR2 APC-Cy7 for 1 h in the dark on ice, followed by CCR2 Alexa Fluor 488 staining without fixation and permeabilization.

### Measuring HIV-1 virion production

To measure the ability of KO cell lines to support the late events in the replication of HIV, we transfected a representative cell line from each of the three groups of KO cell lines (A8 CCL2KO, A7 CCR2KO, and B1 DKO) with an infectious molecular clone DNA of HIV-1_ADA_. At 6 h post-transfection, the media were replaced with fresh media with or without CCL2 (250 ng/mL). At ~36 h post-transfection, supernatants were collected, centrifuged through a 20% sucrose cushion, and the pellets were resuspended. Virus pellets and cell lysates were analyzed via western immunoblots with anti-p24 antibodies as described in the Western blot analysis section.

Furthermore, to study the role of PTAP and LYPX motifs in virus production from the KO cell lines, all nine cell lines were transfected with wild-type HIV-1_ADA_ or its late domain mutants HIV-1_ADA_Δ^LY^ and HIV-1_ADA_^-PTAP^ (2, 46). At 6 h post-transfection, media were replaced with fresh media with or without CCL2 (250 ng/mL). At 24 h post-transfection, supernatants were collected, and the p24 levels were measured via an AlphaLisa detection kit (Perkin Elmer).

### Analysis of soluble and insoluble ALIX levels in parental HeLa cells and KO cells

In order to analyze soluble and F-actin cytoskeleton-bound ALIX, approximately 4 × 10^6^ of parental HeLa cells and all KO cells were seeded and incubated overnight at 37°C. Parental HeLa cells and the CCL2KO cells, B10 and A8, were grown in the presence or absence of CCL2 (250 ng/mL). Parental HeLa cells were also grown in the presence of anti-CCL2 (2.5 μg/mL). CCR2KO (B8, C5, and A7) or DKO cells (B1, B2, and B5) were grown in medium without CCL2 or anti-CCL2. After 48 h, all cells were collected and lysed in a lysis buffer (100 mM HEPES-NaOH, pH 7.5, 142.5 mM KCl, 1% Triton X-100, 5 mM MgCl_2_, and 1:100 dilution of phosphatase inhibitor cocktail I). The lysates were subjected to centrifugation at 20,817 × *g* at 4°C for 15 min. The supernatants (termed S1) were harvested, while the pellets (termed P1), primarily composed of nuclei, cellular debris, and cross-linked actin, were discarded and not investigated further. The supernatant S1 was subjected to centrifugation at 95,000 × *g* at 4°C for 30 min. The supernatant S2 was collected for analysis. The second pellet, P2, was washed 2× with cold PBS and resuspended in 5× sample buffer. The S2 and P2 for all cells were subjected to western immunoblot analysis. Blots were probed with rabbit anti-ALIX (C-terminal) (Sigma-Aldrich, catalog number: SAB4200477-200UL) and anti-β-actin antibodies.

### Construction of ALIX-mGreenLantern expression plasmid and its use in determining ALIX-F-actin colocalization

mGreenLantern sequences (47) were inserted in-frame immediately upstream of the ALIX termination codon in pCI-Neo-FLAG-ALIX via overlap PCR. Primers were designed to amplify ALIX sequences with a 5′ Eco RI restriction site (2,673 bp) and mGreenLantern sequences with 3′ Not I site (747 bp), including homologous sequences at the 3′ end of ALIX and 5′ end of mGreenLantern sequences. The two sequences were brought together in a final PCR containing the two amplicons to generate the full ALIX-mGreenLantern cassette flanked by Eco RI and Not I sites (3,933 bp). All PCR reactions were performed using Q5 High-Fidelity DNA Polymerase (New England Biolabs). The final product was digested by Eco RI and Not I (New England Biolabs) and ligated into a pCINeo backbone to recreate pCI-Neo-FLAG-ALIX-mGreenLantern. The construct was confirmed by restriction digestion and sequencing via both nanopore (Plasmidsaurus, Eugene, OR, USA) and Sanger sequencing.

To study colocalization of pCI-Neo-FLAG-ALIX-mGreenLantern with F-actin in the parental HeLa cells and the KO cells, the parental HeLa cells, B10 CCL2KO, A7 CCR2KO, and B1 DKO cells were seeded on cover slips at a density of 1 × 10^5^ cells per cover slip and incubated overnight at 37°C with 5% CO_2_. The next day, all cells were transfected separately with pCI-Neo-FLAG-ALIX-mGreenLantern (1 µg each) using Lipofectamine 3000 (Invitrogen, ThermoFisher, Waltham, MA, USA). Parental HeLa cells were treated with α-CCL2 (2.5 µg/mL), and KO cells were treated with CCL2 (250 ng/mL) for 24 h. Cells were cooled and washed with ice-cold 10 mM Tris-HCl, pH 7.0, 30 mM KCl, 5 mM MgCl_2_, and 1 mM CaCl_2_ and fixed with 4% Paraformaldehyde (Alfa Aesar) for 15 min at room temperature. Fixed cells were washed three times with the same washing buffer and permeabilized for 15 min with ice-cold 10 mM Tris-HCl, pH 7.0, 60 mM KCl, 125 mM sucrose, and 0.1% Triton X-100. Next, cells were washed as before and treated with Texas Red Phalloidin (Life Technologies) for 30 min at room temperature in the dark. Prolong-Gold (Invitrogen) mounting medium with DAPI was used to overlay glass coverslips. Cells were imaged using a Leica SP8 confocal microscope at a magnification of 63×. Image analysis was performed using NIH Image J software (NIH Image Manual, version 1.61). Up to 40 cells of each type of cell line (HeLa and KO cells) were examined for co-localization of ALIX-mGreenLantern and F-actin via confocal microscopy. Pearson’s coefficient was calculated from all the cells that received the ALIX-mGreenLantern construct via NIH Image J software (NIH Image Manual, version 1.61) and plotted using GraphPad Prism 8.0 (GraphPad Software, San Diego, CA, USA). Pearson’s coefficients were calculated by analyzing each cell separately in confocal sections.

### Testing CCL2 regulation of ALIX in SIV and EIAV budding and release

For SIV and EIAV, parental HeLa cells and A8 CCL2KO cells were seeded in a 10 cm plate at a density of 5 × 10^6^ cells and incubated at 37°C with 5% CO_2_ for 24 h. Cells were transfected with 20 µg of VLP constructs of SIV (pSIV480 [WT] or EIAV [pEV53] or their late domain mutants (pSIV481 [PTAP11-14 LIAL], pSIV482 [Y41S, L48S], or [pEV53 ΔLY]). After 6 h of transfection, cells were treated with CCL2 (250 ng/mL; PeproTech). Media were collected 24 h post-transfection, and the viruses were pelleted through a 20% sucrose cushion. Cells were pelleted and lysed. Both the virus pellet and cell lysates were analyzed via western immunoblot (see Western blot analysis section below).

### Western blot analysis for HIV, SIV, and EIAV virion release

Equal volumes of virions (30 µL) pelleted by sucrose gradient and equal amounts (30 µg) of cell lysates were resolved on either a 15% (for HIV-1) or 4%–20% gradient (for SIV and EIAV) polyacrylamide gels, and proteins were transferred to 0.2 µm nitrocellulose membranes (catalog number, 1620112, BioRad, USA). Membranes were rinsed three times in 1× TBST (Tris-buffered saline, 0.1% Tween-20; 10 min each), then once in 1× TBS (10 min). Blocking was done with Intercept Blocking Buffer (Part No. 927-70001; LI-COR, USA) for HIV or with 5% non-fat dry milk in 1× TPBS buffer for SIV and EIAV, both for 1 h at room temperature. Blots were incubated overnight at 4°C with anti-p24 antibody for HIV (1:1,000; Goat 81, Bleed 000938 from the AIDS and Cancer Virus Program, Frederick National Laboratory for Cancer Research) or with anti-SIV p27 mouse or anti-EIAV CA rabbit antibodies. After washing as before, membranes were incubated for 45 min at room temperature with IRDye 800CW secondary donkey anti-goat antibody (1:5,000; cat # 926-32214, LI-COR, USA) in the case of HIV, goat anti-mouse IgG for SIV, and goat anti-rabbit IgG for EIAV. Following final washes, membranes were scanned using a Gel Doc XR+ (Bio-Rad, USA) or an Odyssey M Imaging System (LI-COR, USA).

### HSV-1 production assay in parental HeLa and A8 CCL2KO HeLa cells

Parental HeLa and A8 CCL2KO cells were grown as described above and infected with HSV-1 strain GS6807 at a multiplicity of infection of 10 in DMEM supplemented with 10% FBS (Peak Serum, Inc.) for 1 h at 37°C, then (using prechilled solutions) washed in phosphate-buffered saline (PBS), treated for 0.5 min with 135 mM NaCl-10 mM KCl-100 mM glycine, pH 3.0 (to inactivate unpenetrated virus), returned to neutral pH by washing twice with PBS, then finally overlaid with prewarmed DMEM-10% FBS. Infected cells were then either collected immediately or incubated for 24 h at 37°C. To determine plaque-forming units (PFU) yields, infected cells were scraped into their medium, sonicated, and the resulting extract was titrated onto preformed Vero cell monolayers as previously described (42).

### DENV growth assay in HeLa cells (WT) and A8 CCL2KO HeLa cells

HeLa and A8 CCL2KO cells were seeded at 0.25 × 10^6^ cells/well in a 12-well plate, cultured overnight, and infected with DENV2 strain 16681 (48) at a MOI of 0.01 IC/well. Culture media were harvested at the indicated time points and titrated by DENV infectious center assay (ICA). DENV ICA was performed on Vero cells similar to the assay described previously for Rubella virus (49). Briefly, Vero cells were seeded at 1.2 × 10^5^ cells/well in 96-well plates and incubated overnight at 37°C with 5% CO_2_. Cells were infected with 10-fold serially diluted virus samples for 4 h. Cells were washed and incubated for 48 h in 100 µL DMEM supplemented with 5% FBS and containing 20 mM NH_4_Cl to prevent secondary infection. Cells were fixed with 4% PFA and stained with the DENV anti-E mAb 4G2 (50, 51). ICAs were developed by staining with an appropriate fluorescent-tagged secondary antibody and quantified by fluorescence microscopy.

### Indirect immunofluorescence assay for HAZV production

Authentic HAZV was acquired from the University of Texas Medical Branch World Reference Center for Emerging Viruses and Arboviruses. Concentrated stocks of HAZV were grown on SW-13 cells purchased from ATCC (CCL-105). Cells were incubated with virus for 4 days at 37°C in DMEM containing 10% FBS and 1% penicillin-streptomycin. Following this, virus-containing supernatant was centrifuged at 28,000 RPM for 2 h at 4°C. Viral pellets were resuspended in NT buffer, aliquoted, and stored at −80°C. To estimate HAZV production, parental HeLa cells, A8 CCL2KO, and A8 CCL2KO treated with CCL2 were seeded as before, and HAZV was added at a dilution of 1:3,000 to each well. Cells were incubated for 5 days at 37°C and inspected for cytopathic effects. The supernatant was harvested for subsequent infection assays. To quantify infection levels, SW-13 cells were seeded in 96-well plates at a density of 1.6 × 10^4^ cells/well. The following day, cells were infected with HAZV-containing supernatant at a dilution of 1:800 for 1 h at 37°C, then 50 mM ammonium chloride-containing medium was added onto the cells, and cells were incubated overnight at 37°C. The following day, the cells were fixed with 4% paraformaldehyde for 5 min, washed, and permeabilized with 0.01% Triton X-100 for 15 min. Cells were then blocked for 30 min with 10% FBS, washed, and stained for 1 h with HAZV reactive polyclonal sera obtained from the United States Army Medical Research Institute of Allergy and Infectious Disease. Cells were then washed three times and stained with a fluorescently tagged anti-mouse Alexa Fluor 594 diluted 1:1,000 in 10% FBS (A-11005). Cells were washed three times and stained for cellular nuclei using Hoechst dye (62249). Plates were imaged using a BioTek Cytation 5 cell imager to quantify the percent Alexa Fluor 594 positivity relative to DAPI using a secondary mask. We also quantified infection levels directly in HeLa cells (untreated and CCL2 treated) and A8 CCL2KO cells (untreated and CCL2 treated) seeded in 96-well plates at a density of 1.6 × 10^4^ cells/well. The cells were infected with HAZV-containing supernatant at a dilution of 1:400 for 1 h at 37°C and then stained with HAZV antibodies as mentioned above.

### Statistical analyses

All experiments were carried out at least three times with two to three experimental replicates for each experiment. The mean of three experiments is presented in our data. Statistical analyses were performed using GraphPad Prism 8.0 (GraphPad Software, San Diego, CA, USA).

## SUPPLEMENTAL MATERIAL

Supplemental material mbio.02241-25-s0001.docx Supplemental methods and figure legends.

Figure S1A mbio.02241-25-s0002.tiff Sequence verification of gene knockout.

Figure S1B mbio.02241-25-s0003.tiff Sequence verification of gene knockout.

Figure S1C mbio.02241-25-s0004.tiff Sequence verification of gene knockout.

Figure S1D mbio.02241-25-s0005.tiff Sequence verification of gene knockout.

Figure S2 mbio.02241-25-s0006.tiff Confirmation of ALIX knockdown in HeLa cells by Western blotting.

ASM does not own the copyrights to Supplemental Material that may be linked to, or accessed through, an article. The authors have granted ASM a non-exclusive, world-wide license to publish the Supplemental Material files. Please contact the corresponding author directly for reuse.
