# Supplementary material for: Influence of CCL2-mediated modulation of ALIX in the budding and replication of viruses from multiple families
Source: mBio. 2025 Sep 25;16(11):e02241-25. doi: 10.1128/mbio.02241-25 (PMC12607790; doi:10.1128/mbio.02241-25)
Supplement: Supplemental material — Supplemental methods and figure legends. [file mbio.02241-25-s0001.docx]

# Supplementary Data

**Methods:**

**Generation and use of ALIX stable knockdown HeLa cells to study the dependence of HIV replication on ALIX**: Stable knockdown of ALIX in HeLa cells was via lentivirus transduction. Briefly, lentiviruses were generated using VSV G and pCMV dR8.91 packaging plasmids and a pLKO.1 expression construct containing an shRNA targeting ALIX (ID: TRCN0000029394). HeLa cells were seeded at a density of 1 x 10^6^ cells/well in a 6-well plate in DMEM media supplemented with 10% FBS and rapamycin (20 µM) to enhance gene transduction efficiency (PMID: 30301809). Two days after ALIX shRNA lentivirus infection of HeLa cells (10ng/10^6^ cells) puromycin selection (2 µg/mL). Following cell expansion, the absence of ALIX expression was verified by western blot.

ALIX* mutant plasmid was generated using splice overlap PCR of the pCI-Neo-FLAG-ALIX plasmid (Addgene:#89859). Mutagenesis primers (FW: 5' - GAAAATC**GCAGCCAAACATTATCAATTC**GCTAGTGGTG -

3', shRNA Rev: 5'CCACTAGC**GAATTGATAATGCTTGGCAGC**GATTTTCA) were used with ALIX flanking primers to amplify and alter the binding site of ALIX targeting shRNA (ID: TRCN0000029394, mutated binding site shown in bold). Fragments were combined via splice overlap PCR using only the flanking primers. The generated fragment was termed ALIX* and was ligated into the pCI-Neo-FLAG-ALIX backbone. After recovery, plasmid identity was confirmed by restriction digest and Sanger sequencing.

For Western blot analysis, confluent cells in 6 cm dishes were collected in lysis buffer (100 mM HEPES, pH 7.5, 142.5 mM KCl, 1% Triton-X-100, 5 mM MgCl2, Phosphatase inhibitor I and II and protease inhibitor. Protein concentrations were verified by Micro BCA Protein Assay kit (ThermoFisher), before equal amounts were loaded on 4-20% Polyacrylamide gels (BioRad). Proteins were transferred onto nitrocellulose membrane by wet transfer, blocked in 5% milk in TBST buffer, and probed with rabbit anti-

ALIX (Proteintech, 124221-AP-1, 1:1000) or mouse anti-GAPDH (Invitrogen, MA515738, 1:10,000) before the addition of anti-rabbit or anti-mouse HRP (1:5000) and chemiluminescent visualization. Note anti- GAPDH antibodies were used after stripping in mild stripping buffer (1.5% (w/v) glycine, 0.1% (w/v) SDS, 1% (v/v) Tween 20).

# Supplementary Figure Legends

**Figure S1**: **Sequence verification of gene knockout**: The three alleles of CCL2 or CCR2 gene (or both in the case of DKO) for one representative line each among the CCL2KO, CCR2KO and DKO lines were aligned with the corresponding genomic sequences of CCL2 or CCR2 using Clustal Omega to highlight the Indels. Each alignment shows the locations of the two corresponding gRNAs used and their orientation as red arrows. A. Alignment of three CCL2KO alleles (#1, #2, #3) derived from **A8 CCL2KO cells** compared to the CCL2 wild-type (WT) sequence (NCBI accession number: NG_012123.1). #1, #2 and #3 correspond to CCL2KO alleles 1, 2 and 3 respectively. B. Alignment of three CCR2KO alleles (#1, #2, #3) derived from **A7 CCR2KO cells** compared to the CCR2 wild-type (WT) sequence (NCBI accession number: NG_021428.1). #1, #2 and #3 correspond to the three alleles of CCR2. C. Alignment of three CCR2KO alleles (#1, #2, #3) derived from **B1 DKO cells** compared to the CCR2 wild-type (WT) sequence (NCBI accession number: NG_012123.1). #1, #2 and #3 correspond to CCR2KO alleles 1, 2 and 3 respectively. D. Alignment of three CCL2KO alleles (#1, #2, #3) derived from **B1 DKO cells** compared to the CCL2 wild-type (WT) sequence (NCBI accession number: NG_021428.1). #1, #2 and #3 correspond to the three CCL2KO alleles.

**Figure S2**: **Confirmation of ALIX knockdown in HeLa cells by Western blotting**. HeLa cells left untreated (UN), treated with a non-targeting lentivirus (Ctrl) or a lentivirus containing ALIX-targeting shRNA (#29394) were collected in lysis buffer. Protein levels were quantified by Micro BCA Kit, before analysis by SDS- PAGE and immunoblotting with polyclonal rabbit anti-ALIX or anti-GAPDH antibodies.
