# Supplementary figures and images for: Influence of CCL2-mediated modulation of ALIX in the budding and replication of viruses from multiple families
Source: mBio. 2025 Sep 25;16(11):e02241-25. doi: 10.1128/mbio.02241-25 (PMC12607790; doi:10.1128/mbio.02241-25)

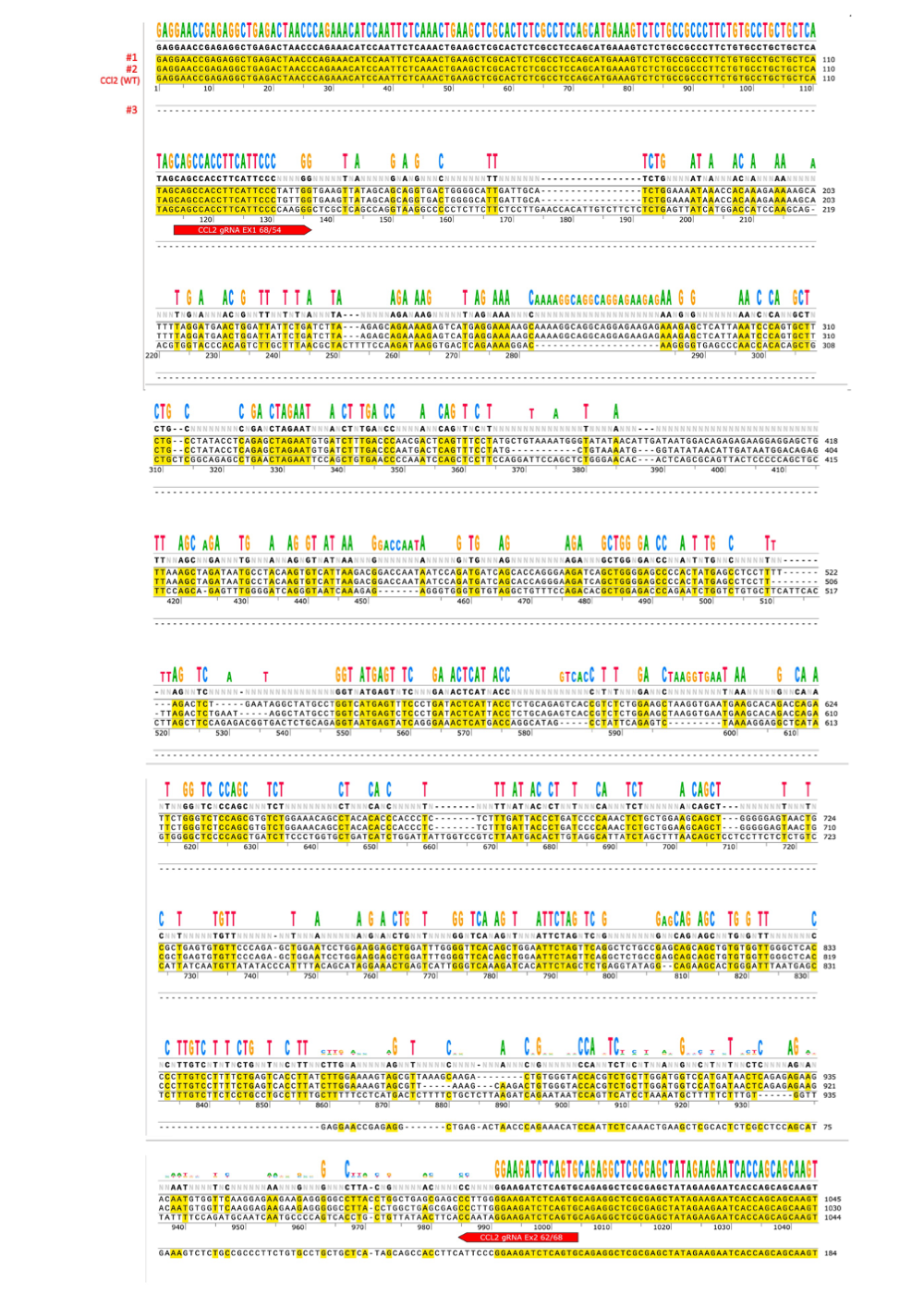

Supplement: Figure S1A — Sequence verification of gene knockout. [file mbio.02241-25-s0002.tiff]

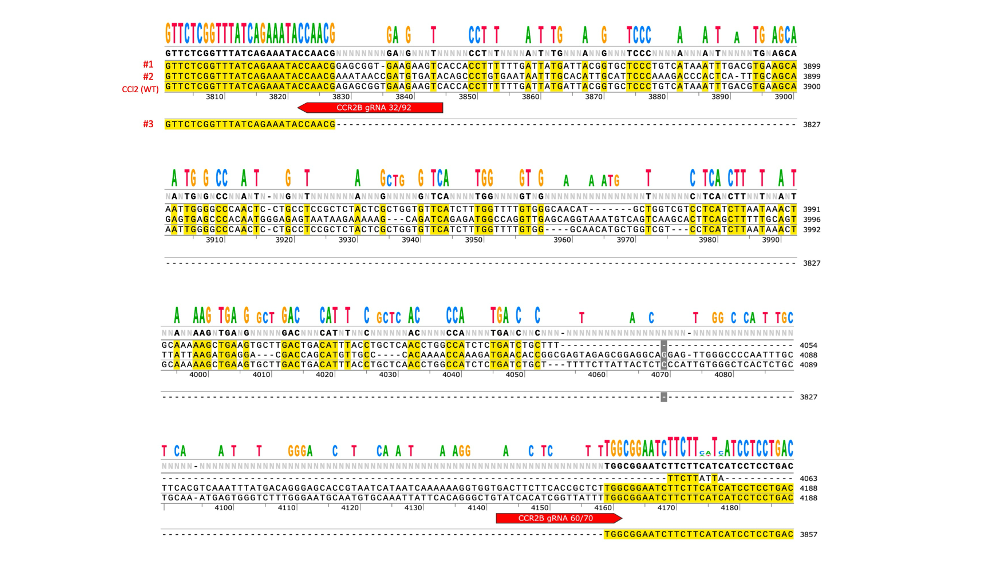

Supplement: Figure S1B — Sequence verification of gene knockout. [file mbio.02241-25-s0003.tiff]

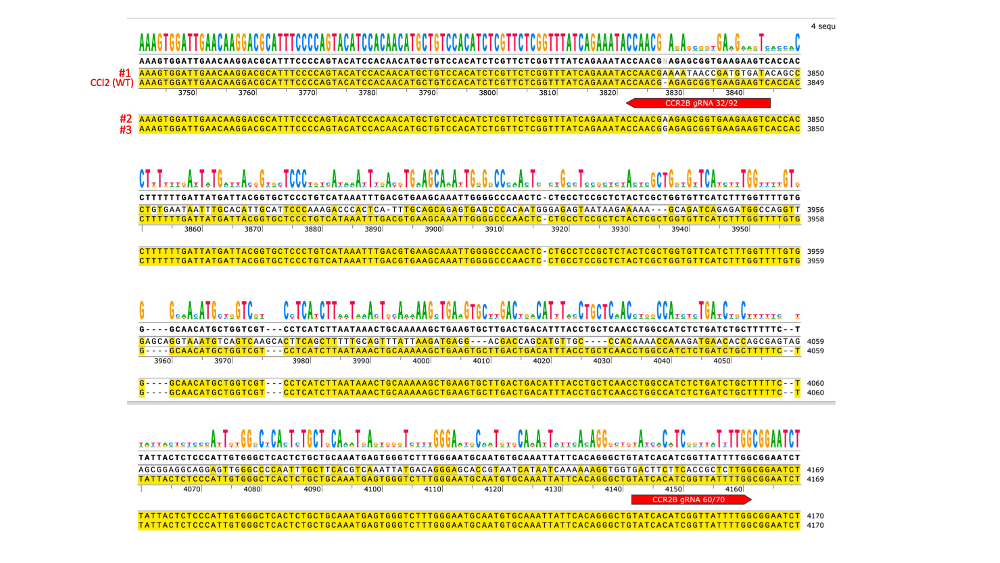

Supplement: Figure S1C — Sequence verification of gene knockout. [file mbio.02241-25-s0004.tiff]

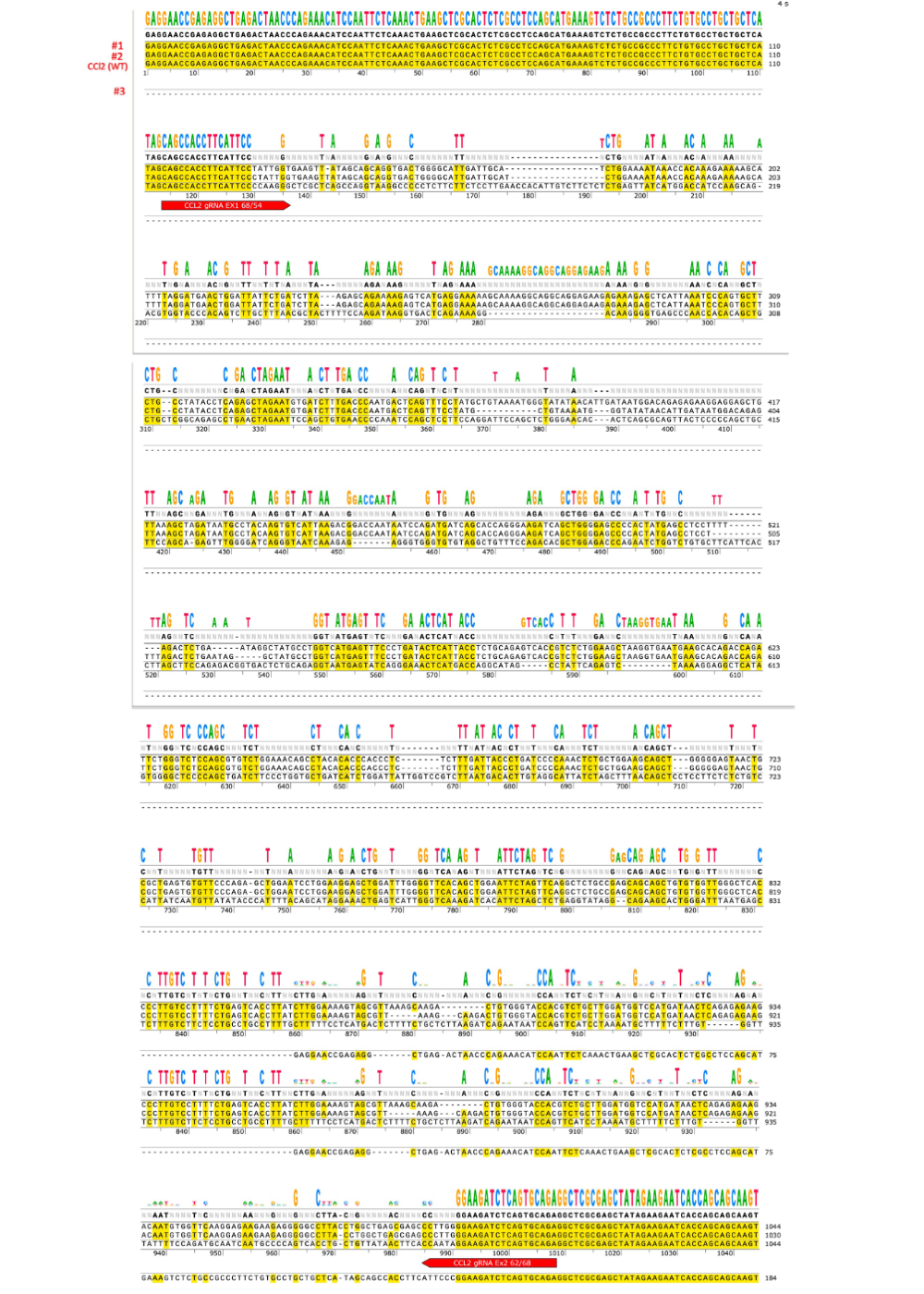

Supplement: Figure S1D — Sequence verification of gene knockout. [file mbio.02241-25-s0005.tiff]

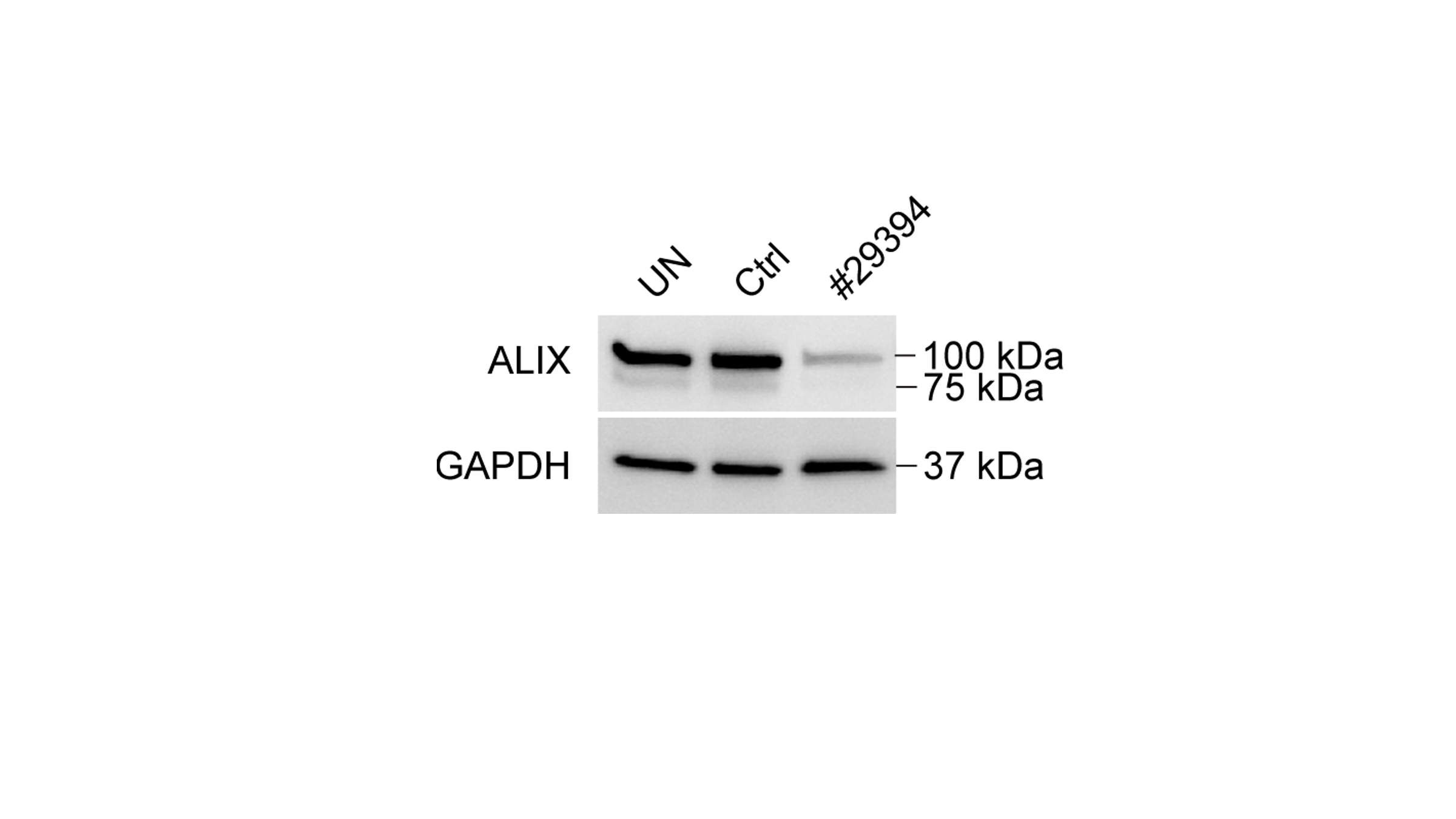

Supplement: Figure S2 — Confirmation of ALIX knockdown in HeLa cells by Western blotting. [file mbio.02241-25-s0006.tiff]
